# Human midbrain dopaminergic neuronal differentiation markers predict cell therapy outcomes in a Parkinson’s disease model

**DOI:** 10.1172/JCI156768

**Published:** 2022-07-15

**Authors:** Peibo Xu, Hui He, Qinqin Gao, Yingying Zhou, Ziyan Wu, Xiao Zhang, Linyu Sun, Gang Hu, Qian Guan, Zhiwen You, Xinyue Zhang, Wenping Zheng, Man Xiong, Yuejun Chen

**Affiliations:** 1 Institute of Neuroscience, Key Laboratory of Primate Neurobiology, CAS Center for Excellence in Brain Science and Intelligence Technology, Chinese Academy of Sciences, Shanghai, China.; 2 University of Chinese Academy of Sciences, Beijing, China.; 3 State Key Laboratory of Medical Neurobiology and MOE Frontiers Center for Brain Science, Institutes of Brain Science, Fudan University, Shanghai, China.; 4 Shanghai Center for Brain Science and Brain-Inspired Intelligence Technology, Shanghai, China.

**Keywords:** Neuroscience, Stem cells, Parkinson disease, Stem cell transplantation

## Abstract

Human pluripotent stem cell–based (hPSC-based) replacement therapy holds great promise for the treatment of Parkinson’s disease (PD). However, the heterogeneity of hPSC-derived donor cells and the low yield of midbrain dopaminergic (mDA) neurons after transplantation hinder its broad clinical application. Here, we have characterized the single-cell molecular landscape during mDA neuron differentiation. We found that this process recapitulated the development of multiple but adjacent fetal brain regions including the ventral midbrain, the isthmus, and the ventral hindbrain, resulting in a heterogenous donor cell population. We reconstructed the differentiation trajectory of the mDA lineage and identified calsyntenin 2 (CLSTN2) and protein tyrosine phosphatase receptor type O (PTPRO) as specific surface markers of mDA progenitors, which were predictive of mDA neuron differentiation and could facilitate high enrichment of mDA neurons (up to 80%) following progenitor cell sorting and transplantation. Marker-sorted progenitors exhibited higher therapeutic potency in correcting motor deficits of PD mice. Different marker-sorted grafts had a strikingly consistent cellular composition, in which mDA neurons were enriched, while off-target neuron types were mostly depleted, suggesting stable graft outcomes. Our study provides a better understanding of cellular heterogeneity during mDA neuron differentiation and establishes a strategy to generate highly purified donor cells to achieve stable and predictable therapeutic outcomes, raising the prospect of hPSC-based PD cell replacement therapies.

## Graphical abstract

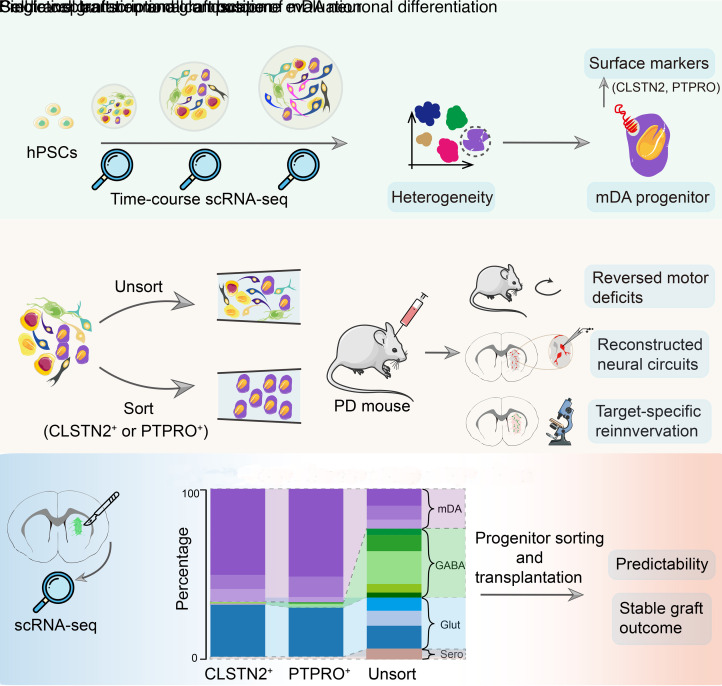

## Introduction

Stem cell–based replacement therapy has been one of the most promising strategies for treating Parkinson’s disease (PD) (1–4). Human pluripotent stem cells (hPSCs), including human embryonic stem cells (hESCs) and human induced pluripotent stem cells (hiPSCs), represent an essentially unlimited source of cells and have been used to produce transplantable midbrain dopaminergic (mDA) progenitors via in vitro differentiation (5–7). hiPSC- or hESC-derived dopamine neurons exhibited long-term survival when implanted into rodent (5, 6, 8) or nonhuman primate models of PD (9–11), resulting in behavioral improvements. Encouraged by these findings, clinical trials evaluating hPSC-based PD cell therapies were launched in several countries in Europe as well as in Japan, Australia, China, and the United States (12–15).

However, challenges remain. hPSC-derived donor cells are highly heterogeneous, as they contain variety of cell types with different degrees of maturity and fate potentials. As a result, the proportion of target neurons (mDA neurons) in the transplants is typically low, and there is high variability among different batches and cell lines (10, 16). In preclinical studies using mice or rats, no more than 10% of the grafted cells were tyrosine hydroxylase–positive (TH^+^) mDA neurons (17, 18). The identities of the remaining cells within the grafts (i.e., off-target cells) have not been fully understood. However, contamination of serotonin neurons is considered a major cause of graft-induced dyskinesia, and poorly differentiated cells may result in graft overgrowth or teratoma formation after transplantation (13, 19–21). These factors — a low percentage of DA neurons, cellular heterogeneity, and uncharacterized graft composition — represent major obstacles for the broad application of PD cell therapy in the clinic (9, 11, 16). What remains largely unknown is how the mDA lineage is specified during differentiation, what the cellular identities of those unwanted cells (non-mDA cells) are in the donor cells and how they are generated, and whether it is possible to produce homogeneous donor cells composed of purified target cells and depleted off-target cell types to ensure stable and predictable graft outcomes.

To address these questions, we constructed a comprehensive, single-cell transcriptional atlas of mDA neuron differentiation from hPSCs. We found that the process of mDA-directed differentiation resembles the development of adjacent fetal brain regions including ventral midbrain, midbrain-hindbrain boundary (MHB, also known as isthmus), and ventral hindbrain, which explains the cause of cellular heterogeneity and the origin of off-target cells. We specifically reconstructed the differentiation trajectory of mDA lineage and discovered stage-dependent surface markers representing early or late mDA progenitors, which were predictive of mDA neuron fate and could give rise to highly enriched mDA neurons following cell sorting and transplantation. We further showed that surface marker–sorted progenitors exhibited a higher therapeutic potency in correcting motor function deficits in a mouse model of PD. Most important, mDA grafts from these surface marker–sorted progenitors lacked contaminating neurons that might underlie untoward clinical outcomes (e.g., graft-induced dyskinesia), and different marker-sorted grafts had strikingly consistent cell-type composition. Our study provides a comprehensive understanding of cellular identities and lineage trajectories during mDA neuron differentiation and demonstrates its application in determining the means for controlling the variability and heterogeneity of donor cells to achieve improved therapeutic outcomes for PD cell therapy.

## Results

### Time-course single-cell RNA-Seq reveals cellular heterogeneity during differentiation of mDA neurons from hPSCs.

**Figure 1:**
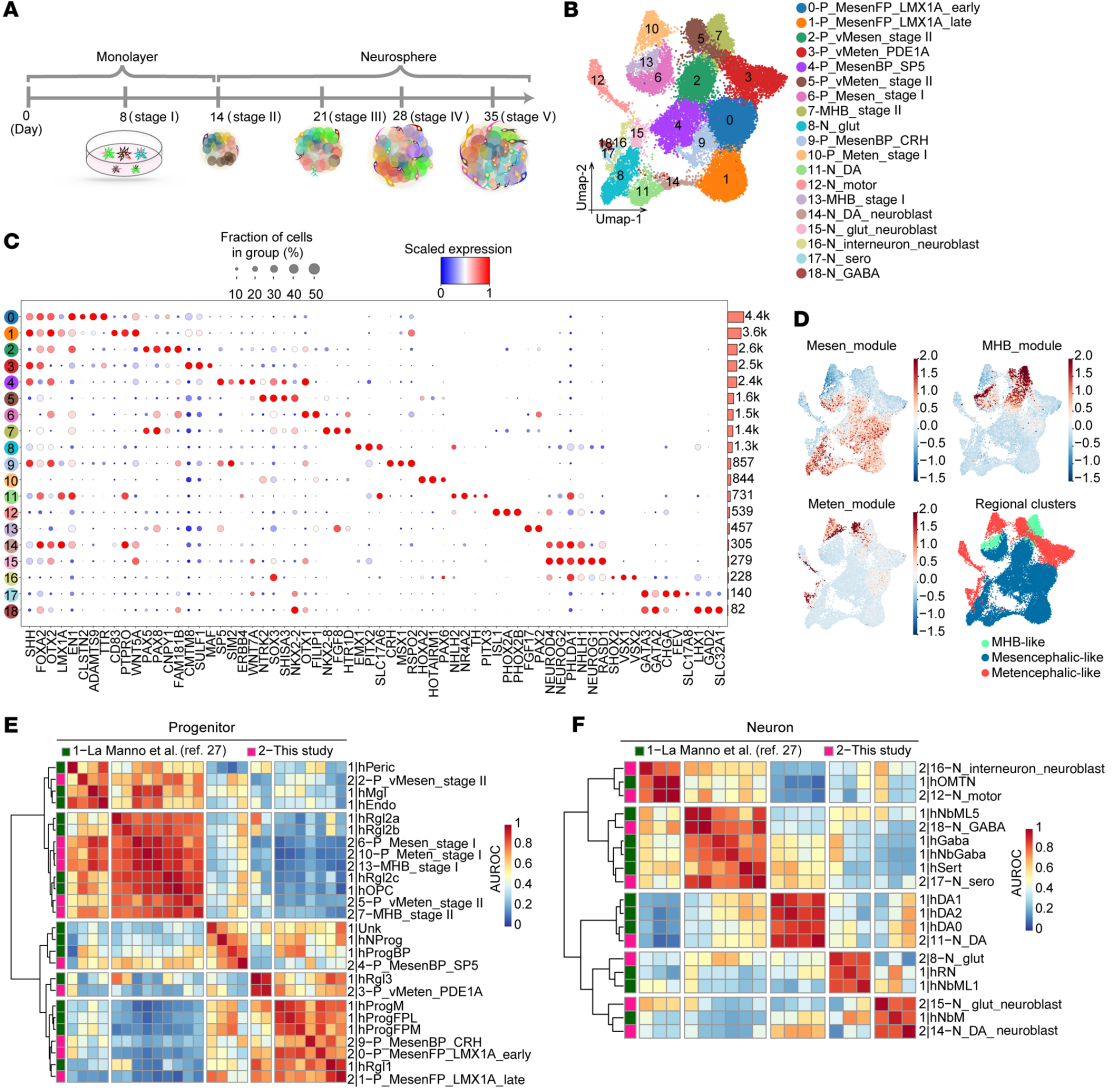
Time-course single-cell RNA-Seq reveals cellular heterogeneity during differentiation of mDA neurons from hPSCs. (**A**) Schematics of in vitro cell differentiation process for scRNA-Seq. (**B**) Visualization of clustering results of merged data sets from all stages using uniform manifold approximation and projection (UMAP). (**C**) Dot plot showing classical markers of floor plate and representative markers (column) for each cell type (row). Mean gene expression has been scaled between 0 and 1. Horizontal bars denote the number of cells in each cluster. Cell-type labels are used as UMAP clusters in **B**. The dot color scale represents average expression levels, and dot size represents the fraction of cells in a group. (**D**) Regional gene module expression and regional annotation of time-course scRNA-Seq data. See Supplemental Methods for the module gene lists used to calculate the gene expression scores. (**E** and **F**) Heatmaps of area under the receiver operating characteristic (AUROC) scores between progenitor (**E**) and neuron (**F**) clusters in this study and data from a public data set (27). P, progenitor; N, neuron; vMesen, ventral mesencephalic; vMeten, ventral metencephalic; MesenFP, mesencephalic floor plate; MesenBP, mesencephalic basal plate.

Human mDA neurons can now be readily differentiated from hPSCs according to established protocols including ours (8, 22), however, the dynamic changes in cellular composition and gene expression during differentiation are not yet fully understood. We collected cells for single-cell RNA-Seq (scRNA-Seq) at the end of 5 consecutive stages of differentiation of mDA neurons from hPSCs (see Methods and Figure 1A). After the removal of low-quality cells, a total of 25,776 cells were used for analysis. We identified a total of 19 distinct cell clusters based on known marker genes related to regional brain identities and neural developmental states (Figure 1, B and C, and Supplemental Table 1; supplemental material available online with this article; https://doi.org/10.1172/JCI156768DS1). These cell clusters included mDA progenitors in different developmental states (clusters 0 and 1), which were characterized by the expression of canonical mDA progenitor markers including *LMX1A*, *FOXA2*, *EN1*, and *OTX2* (23–26), and mDA neurons expressing *TH* and *PITX3* (cluster 11) (Figure 1, B and C). In addition to dopaminergic lineage cells, we observed various non-mDA neurons including serotoninergic neurons (cluster 17), GABAergic neurons (cluster 18), motor neurons (cluster 12), and glutamatergic neurons (cluster 8) (Figure 1, B and C). We also detected various non-midbrain progenitor populations including MHB-like cells (clusters 7 and 13) and metencephalic progenitors (clusters 3, 5, and 10) (Figure 1, B and C). Other cell populations included different types of non-mDA mesencephalic progenitors (clusters 4 and 9) and neuroblasts (clusters 15 and 16). By calculating gene modules that represent mesencephalic, metencephalic, or MHB cells (Supplemental Methods), we found that all of the differentiated cells could be classified into these 3 regional identities (Figure 1D). These results suggest that the differentiation of mDA neurons from hPSCs produced a large number of heterogeneous cell populations, with regional identities spanning from the midbrain to the hindbrain.

We further validated the identities of the cells generated during mDA-directed differentiation. We found that expression of the mouse homologs of the marker genes of the annotated human cell clusters, including mDA progenitors, various non-mDA progenitors, and different types of neurons, were specifically expressed in the developing mouse ventral midbrain, MHB, and ventral hindbrain at E11.5 and E13.5 (Supplemental Figure 1, A and B). Then, we compared our data with a public scRNA-Seq data set from human fetal midbrain using MetaNeighbor (Supplemental Methods). The clustering hierarchical dendrogram between these 2 data sets and the similarity score suggested that the cell types generated in our protocol resembled their in vivo counterparts (Figure 1, E and F). For instance, our annotated mDA progenitors (P_MesenFP_LMX1A_Late) were transcriptionally similar to hRgl1, which was reported to be a potential human mDA progenitor (Figure 1E) (27). Also, the hPSC-derived neuronal cell types including mDA neurons, serotonin neurons, and motor neurons have the transcriptomic profiles similar to that of their endogenous counterparts in the human fetal brain (Figure 1F). Altogether, our data revealed the high heterogeneity of cell populations generated during mDA neuron differentiation from hPSCs and that the molecular features of these cells resembled those of cells in the developing brain.

### The process of mDA neuron differentiation recapitulates the development of adjacent fetal brain regions including the ventral midbrain, the isthmus, and the ventral hindbrain.

**Figure 2:**
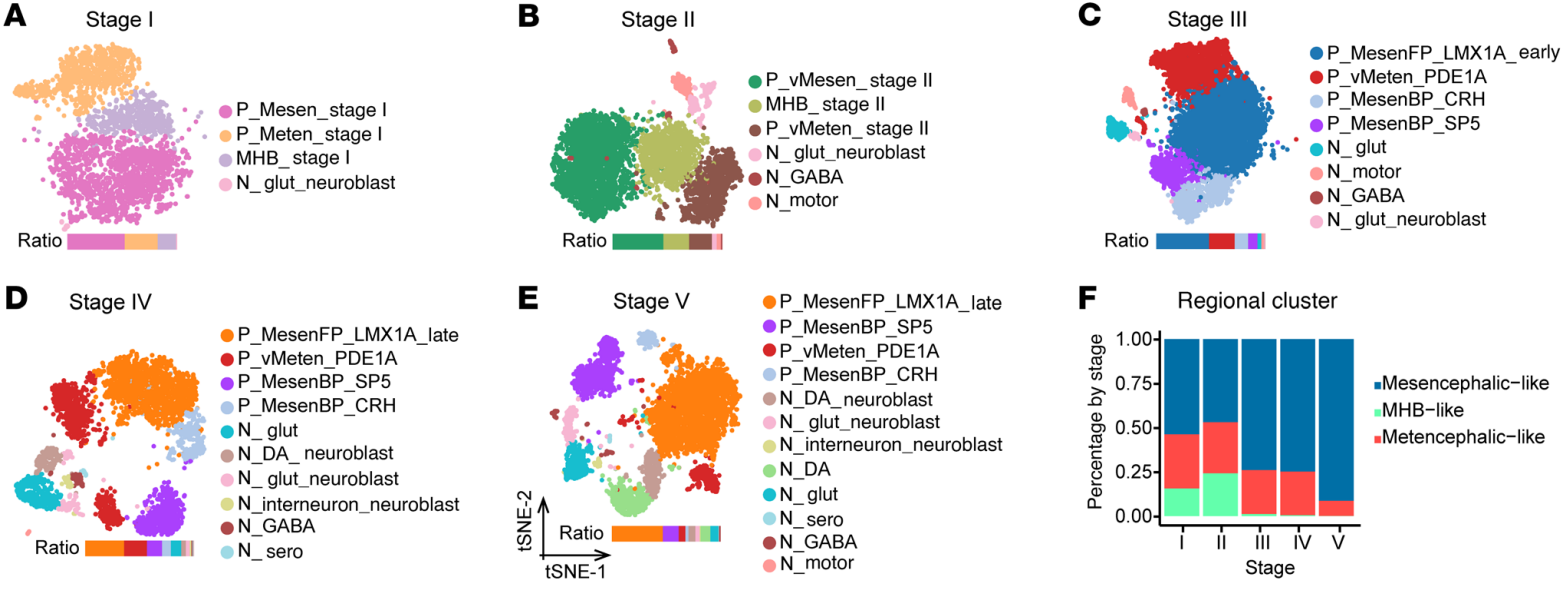
Cell-type composition at each stage of mDA neuron differentiation. (**A–E**) Visualization of clustering results for each stage using *t*-distributed stochastic neighbor embedding (*t*-SNE). (**F**) Change in the percentage of regional clusters by stage. The MHB-like cluster mainly emerged at the early patterning stage (stages I and II).

**Figure 3:**
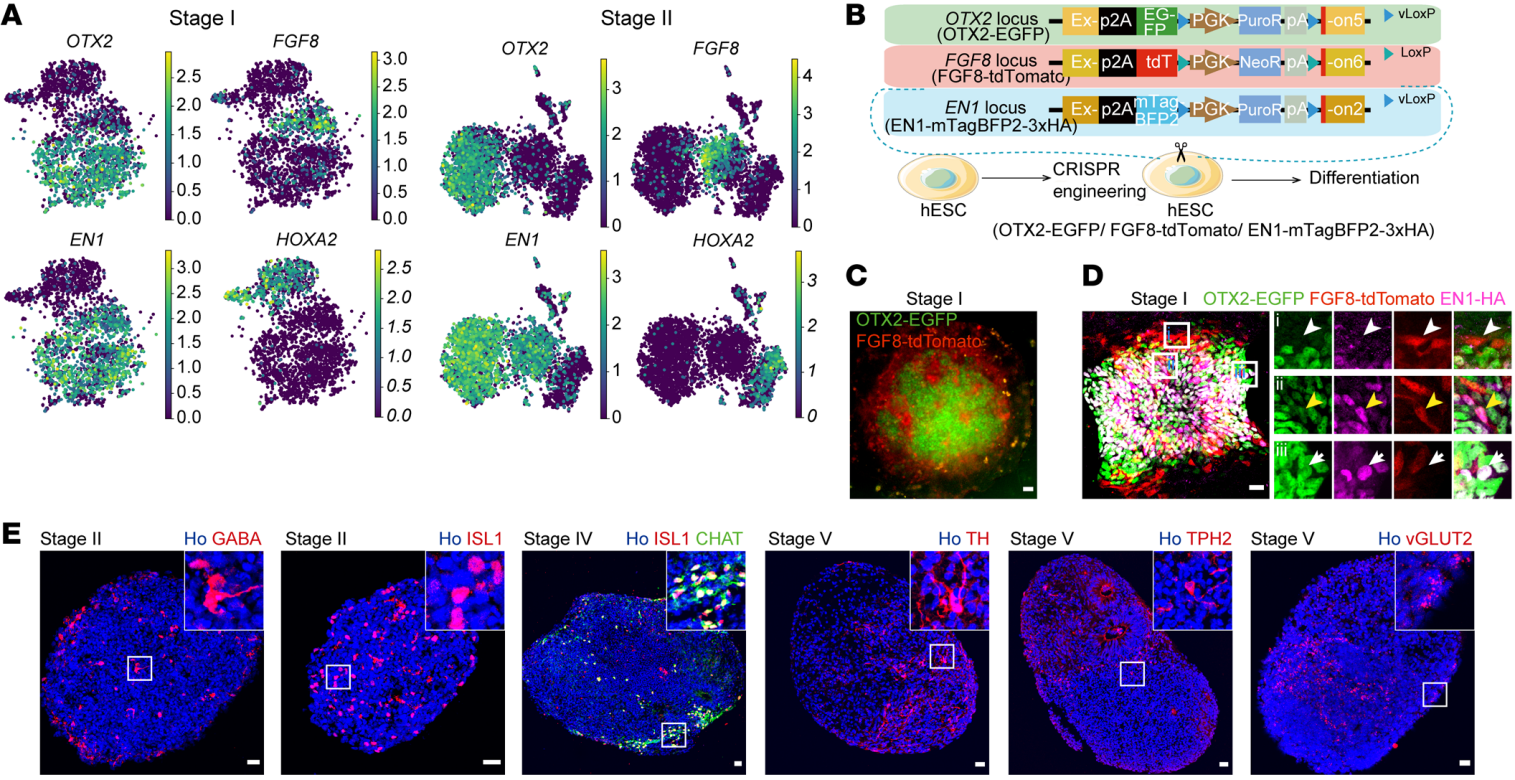
The process of mDA neuron differentiation recapitulates the development of adjacent fetal brain regions including the ventral midbrain, the isthmus, and the ventral hindbrain. (**A**) Stage I or II scRNA-Seq clusters of cells showing expression of *OTX2*, *FGF8*, *EN1*, and *HOXA2* genes. (**B**) OTX2/FGF8/EN1 reporter cell line diagram. (**C**) Typical colony of stage I EGFP/tdT cells by live imaging. (**D**) Typical colony of stage I EGFP/tdT cells by immunostaining for EGFP/tdT/HA-tag. The white arrowhead, yellow arrowhead, and white arrow indicate an FGF8-tdT^+^ cell, FGF8-tdT^+^EN1-HA^+^ cell, and OTX2-EGFP^+^EN1-HA^+^ cell, respectively. Scale bars: 50 μm (**C**) and 25 μm (**D**). Original magnification, ×20 (enlarged insets in **D**). (**E**) Typical neurospheres immunostained for neuronal markers at distinct stages. VGLUT2 was validated by RNA-FISH (RNAScope) and the others by antibodies. Scale bars: 25 μm.

To explore the dynamic changes in cellular heterogeneity along with differentiation, we performed clustering analysis of cells at each differentiation stage and calculated the cell-type composition at each stage on the basis of regional identities, which revealed that mesencephalic-like and metencephalic-like cells were present at all differentiation stages, whereas MHB-like cells temporally appeared only at early stages (I, II) (Figure 2). Interestingly, we found that differentiated cells had molecular features with distinct regional identities, including mesencephalic cells expressing *OTX2* and *EN1*, metencephalic cells expressing *HOXA2*, and MHB cells expressing *FGF8*, as early as 8 days after differentiation (stage I), suggesting early regional fate determination of these cells (Figure 3A). The mesencephalic and metencephalic cells became ventralized 14 days after differentiation (stage II), as revealed by the expression of the basal plate and floor plate marker *FOXA2* (Figure 1C and Supplemental Figure 1A). We found that the cellular diversity of the culture increased along with mDA neuron differentiation. For instance, mesencephalic progenitors were further divided into 3 progenitor subclusters at the middle or late stage of differentiation (stage III or V), while we observed only 1 mesencephalic progenitor cluster at the early stages of differentiation (stages I–II) (Figure 2, A–E). mDA progenitors characterized by the expression of *LMX1A*, *EN1*, *OTX2*, and *FOXA2* (Figure 1C and Figure 2C) appeared at the middle stage of differentiation (stage III, cluster P_MesenFP_LMX1A_Early). mDA neuroblasts expressing *LMX1A* and *NEUROG2*, and mDA neurons expressing *TH* and *PITX3* emerged at the late stages of differentiation (stages IV and V) (Figure 1C and Figure 2, D and E). For other neuron types, serotonin neurons (N_Sero) appeared until the late stages (IV–V) (Figure 1C and Figure 2, D and E), whereas motor neurons (N_Motor), glutamate neurons (N_Glut), and GABA neurons (N_GABA) were present at early-to-mid stages or across all of the stages (Figure 2, A–E).

To validate early regional patterning during mDA neuron differentiation, we introduced reporter constructs specific for 3 canonical regional markers including *OTX2* (expressed in the prosencephalon and mesencephalon), *EN1* (expressed in the mesencephalon, MHB, and rostral metencephalon), and *FGF8* (expressed in the MHB) into hPSCs. We named this triple-reporter hPSC line H9-OTX2-EGFP/FGF8-tdTomato (tdT)/EN1-mTagBFP2-3xHA (Figure 3B and Supplemental Figure 1C). We observed FGF8-tdT^+^ cells by live-cell imaging and immunofluorescence staining 8 days after differentiation (stage I) **(**Figure 3, C and D). FGF8-tdT^+^ cells persisted at stage II (Supplemental Figure 1, D and E), but diminished at the late stage of differentiation (Supplemental Figure 1F). These results were consistent with our scRNA-Seq data, further confirming the early transient presence of MHB-like cells during mDA neuron differentiation (Figure 2F). This phenotype is very similar to the transient presence of MHB during early fetal brain development (28). Immunofluorescence staining also confirmed the presence of mesencephalic cells (OTX2-EGFP^+^EN1-HA^+^, white arrows in Figure 3D) in the culture at the early stages of mDA neuron differentiation. These data confirmed early regional patterning during differentiation (Figure 3, C and D, and Supplemental Figure 1, D and E). We also verified the existence of various mid-stage progenitors and types of neurons at different differentiation stages (Figure 3E and Supplemental Figure 1, G and H). Notably, we observed a considerable proportion of mDA neurons expressing *NR4A2*, *TH*, and *PITX3* at the terminal differentiation stage (Supplemental Figure 1, H and I).

Altogether, by constructing the single-cell molecular atlas during mDA neuron differentiation, we demonstrate that the process of hPSC-based mDA neuron differentiation recapitulated the in vivo development of adjacent fetal brain regions including ventral midbrain, isthmus, and ventral hindbrain regions. In particular, the mid-hindbrain regional identities of the cells were established at very early stages of differentiation, while the specification of mDA progenitors and neurons occurred at the mid-to-late stage of differentiation. These findings also reveal the source of off-target cells, that is, the cell populations generated along with ventral midbrain floor plate progenitors (mDA progenitors), a result of the in vitro neural differentiation that simulates in vivo neurodevelopment. These cell populations included metencephalic progenitors (cluster 3 expressing *CMTM8* and *SULF1*) and non-mDA mesencephalic progenitors (cluster 4 expressing *SP5* and *SIM2* and cluster 9 expressing *CRH* and *MSX1*) (Figure 1C and Figure 2, A–E).

### Reconstruction of the single-cell trajectory of mDA neuron differentiation reveals dynamic and characteristic transcriptional profiles along lineage specification.

**Figure 4:**
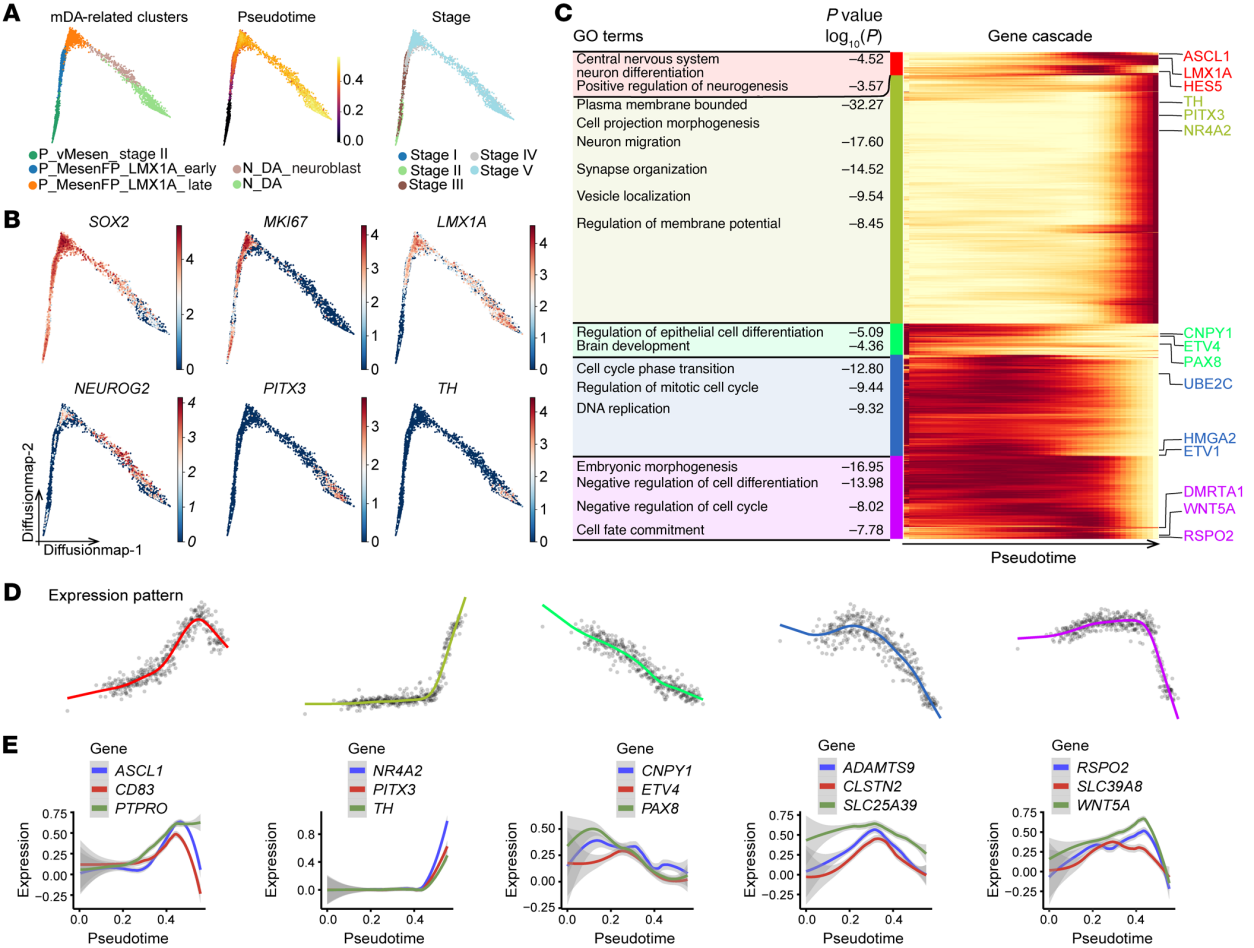
Reconstruction of single-cell trajectory of mDA neuron differentiation reveals a dynamic and characteristic lineage-specific transcriptional profile. (**A**) Visualization using diffusion map embeddings by mDA-related clusters, pseudotime, and stage. (**B**) Typical marker expression on a diffusion map. (**C**) Enriched GO terms for each gene cluster (left) and gene expression cascade (right) during mDA differentiation. Heatmap shows selected gene expression along pseudotime. Expression is displayed as the mean expression of groups of 5 cells and was smoothed using a spline curve and scaled to the maximum observed expression (low expression in yellow, high expression in red). The colored label along the left side of the heatmap identifies the gene cluster. (**D**) Mean expression profiles for each gene cluster. The colors of the spline curves correspond to the gene cluster colors in **C**. (**E**) Expression of selected genes for each gene cluster. The curve represents the mean expression of the gene, and the standard error of the mean is shown as a gray band.

To define the transcriptional path of mDA neuron differentiation from hPSCs, we subjected our scRNA-Seq data sets to pseudotime (URD) and single-cell trajectory (scVelo) analyses (29, 30). First, by calculating the RNA velocity stream and the gene-shared latent time of all cell types, we found that progenitors were in an early latent time state, whereas neuroblasts and neurons were in a late one (Supplemental Methods and Supplemental Figure 2A). Diffusion map–based embeddings of pseudotime calculated on mDA-related clusters including P_MesenFP_LMX1A_Early, P_MesenFP_LMX1A_Late, P_vMesen_Stage II, N_DA and N_DA_Neuroblast showed that the cells from these clusters were ordered from early mesencephalic floor plate progenitors to mature mDA neurons, or from early differentiation stage to late differentiation stage, following the pseudotime (Figure 4A). As expected, several known markers of neural stem cells (*SOX2* and *MKI67*), mDA progenitors (*LMX1A*), neurogenic cells (*NEUROG2*), and mDA neurons (*PITX3* and *TH*) were expressed at early and late pseudotime points, respectively (Figure 4B). Of note, the percentage of dividing cells in mDA-related clusters decreased over time, demonstrating that mDA progenitors gradually exited the cell cycle during differentiation (Supplemental Figure 2B).

To depict the temporal dynamics of gene expression of mDA-related clusters during differentiation, we selected genes whose expression highly varied along the pseudotime (Supplemental Methods). Five gene clusters representing distinct expression patterns were identified from 1074 varying genes. Further exploration by Gene Ontology (GO) analysis of these 5 gene clusters revealed that the early-off/late-on genes were mainly involved in mDA neurogenesis and maturation processes (Figure 4C, second block of GO terms [yellow-green block]). These genes included *NR4A2, PITX3*, and *TH*, which were greatly upregulated at the late pseudotime point (Figure 4, D and E, second panel). We also identified an early-on/late-off gene cluster (Figure 4E, third panel) including *CNPY1, ETV4*, and *PAX8* (Figure 4C, third block of GO terms [bright green block]). The expression of *CNPY1* and *PAX8* in the donor progenitor cells has been reported to be positively correlated with the yield of mDA neurons after transplantation (16), indicating putative roles of these genes in the early fate determination of mDA progenitors. The remaining 3 gene clusters were identified as “mid-on” clusters (Figure 4, D and E, first, fourth and fifth panels). These genes include *CD83*, protein tyrosine phosphatase receptor type O (*PTPRO*), calsyntenin 2 (*CLSTN2*), *SLC25A39*, *WNT5A*, and *RSPO2*, which were temporally or highly expressed in the middle of the pseudotime trajectory (Figure 4E, first, fourth and fifth panels). Although the functions of these genes during mDA neuron differentiation are not known, some of them, such as *WNT5A* and *RSPO2*, were previously reported to regulate mDA neurogenesis in vivo or in vitro (31–33), suggesting potential roles of these “mid-on” genes in regulating mDA neuron differentiation. In addition, we also depicted the temporal and dynamic expression of specific transcription factors potentially involved in the fate determination of mDA neurons (Supplemental Figure 2, C and D) or various ligands/receptors functioning in ventral midbrain patterning along pseudotime (Supplemental Methods and Supplemental Figure 2, E and F) (34). Together, by reconstruction of a single-cell trajectory of hPSC-based mDA neuron differentiation and pseudotime analysis, we reveal dynamic and characteristic transcriptional profiles along mDA neuron lineage specification.

### CLSTN2 and PTPRO are identified as specific surface markers for early and late mDA progenitors, respectively.

**Figure 5:**
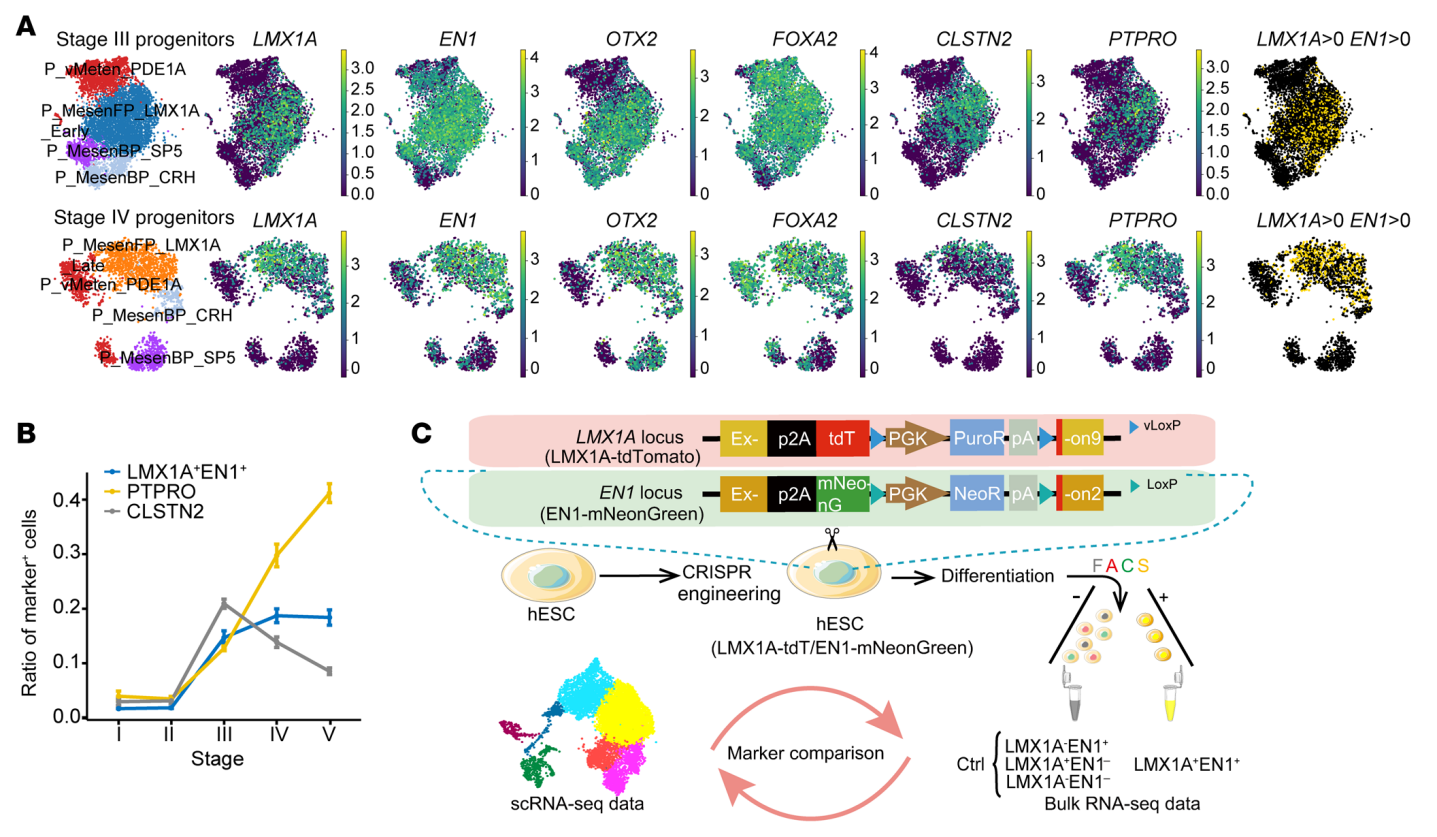
CLSTN2 and PTPRO are identified as specific surface markers for early and late mDA progenitors, respectively. (**A**) Progenitor clusters; expression of mDA progenitor marker genes (*LMX1A*, *EN1*, *OTX2*, and *FOXA2*) and of 2 identified surface marker genes (*CLSTN2*, *PTPRO*); and annotated LMX1A^+^EN1^+^ cells (*LMX1A* unique molecular identifier [UMI] counts >0 and *EN1* UMI counts >0) on UMAP embeddings of stage III and stage IV progenitors. (**B**) Selected marker-positive cell ratio for a random set of cells (10% of cells from each stage). Data represent the mean ± SD. See Supplemental Methods for details. (**C**) Dual-reporter cell line diagram and schematics of joint analysis of bulk RNA-Seq and scRNA-Seq data. Ctrl, control.

Having resolved the heterogeneity of cell populations generated during mDA neuron differentiation and the molecular basis underlying mDA lineage specification, we next explored whether it is possible to distinguish and isolate mDA progenitors from heterogeneous donor cells by specific markers, so as to purify mDA progenitors and eliminate unwanted cells, thereby producing highly enriched donor cells. We performed differential gene expression analysis on the mDA progenitor cluster at stage III (cluster P_MesenFP_LMX1A_Early) and stage IV (cluster P_MesenFP_LMX1A_Late), respectively, which was calculated by the “FindAllMarkers” function in Seurat (Supplemental Table 2). We searched the top 50 differentially expressed genes (DEGs) with a defined threshold (log_2_ fold change of the average expression between the 2 groups >0.5 and the percentage of cells in which the gene was detected in the non-mDA progenitor group *<*0.2). Surprisingly, we identified 2 surface markers, *CLSTN2* (at stage III) and *PTPRO* (at stage IV), which were specifically expressed in mDA progenitors characterized by the coexpression of *LMX1A*, *EN1*, *OTX2*, and *FOXA2* (Figure 5A and Supplemental Figure 3A). Interestingly, both *CLSTN2* and *PTPRO* were categorized in the “mid-on” gene cluster in the single-cell gene expression pattern analysis (Figure 4, D and E, first and fourth panels), indicating expression of both genes in mDA progenitors along with the differentiation process. By calculating the ratio of *CLSTN2*^+^ or *PTPRO*^+^ cells at each stage (Supplemental Methods), we found that the *CLSTN2*^+^ population peaked at stage III (Figure 5B, gray curve), whereas the *PTPRO*^+^ population emerged at stage III and gradually increased over time (Figure 5B, yellow curve). Of note, we detected the expression of *PTPRO* in non-mDA progenitors at the late stage of differentiation (stage V; Supplemental Figure 3A), indicating that *PTPRO* was expressed specifically in mDA progenitors only at certain stages. We observed similar temporal dynamic trends in the averaged expression of these 2 marker genes using scRNA-Seq pseudo-bulk analysis (Supplemental Figure 3B and Supplemental Methods).

In the developing mouse brain, *LMX1A* is expressed between the floor plate of the diencephalon and the mesencephalon, whereas *EN1* is expressed from the caudal mesencephalon to the rostral metencephalon. The brain regions with overlapping expression of these 2 markers could be designated as the mesencephalic floor plate, where mDA neurons originate (23–26, 35, 36). As expected, we found that *LMX1*A and *EN1* double-positive cells (LMX1A^+^EN1^+^) were exclusively located in mDA progenitor clusters (Figure 5A). We therefore chose the combination of *LMX1A* and *EN1* as better transcription factor markers representing mDA progenitors (Figure 5C). The *LMX1A*^+^*EN1*^+^ cell population initially emerged at stage III, peaked at stage IV, and showed sustained expression until stage V (Figure 5B, blue curve). We created a dual-reporter hPSC line to illuminate authentic mDA progenitors by inserting the fluorescent proteins tdT and mNeonGreen into the *LMX1A* and *EN1* loci, respectively (LMX1A-tdT/EN1-mNeonG hPSCs) (Figure 5C and Supplemental Figure 3, C and D).

**Figure 6:**
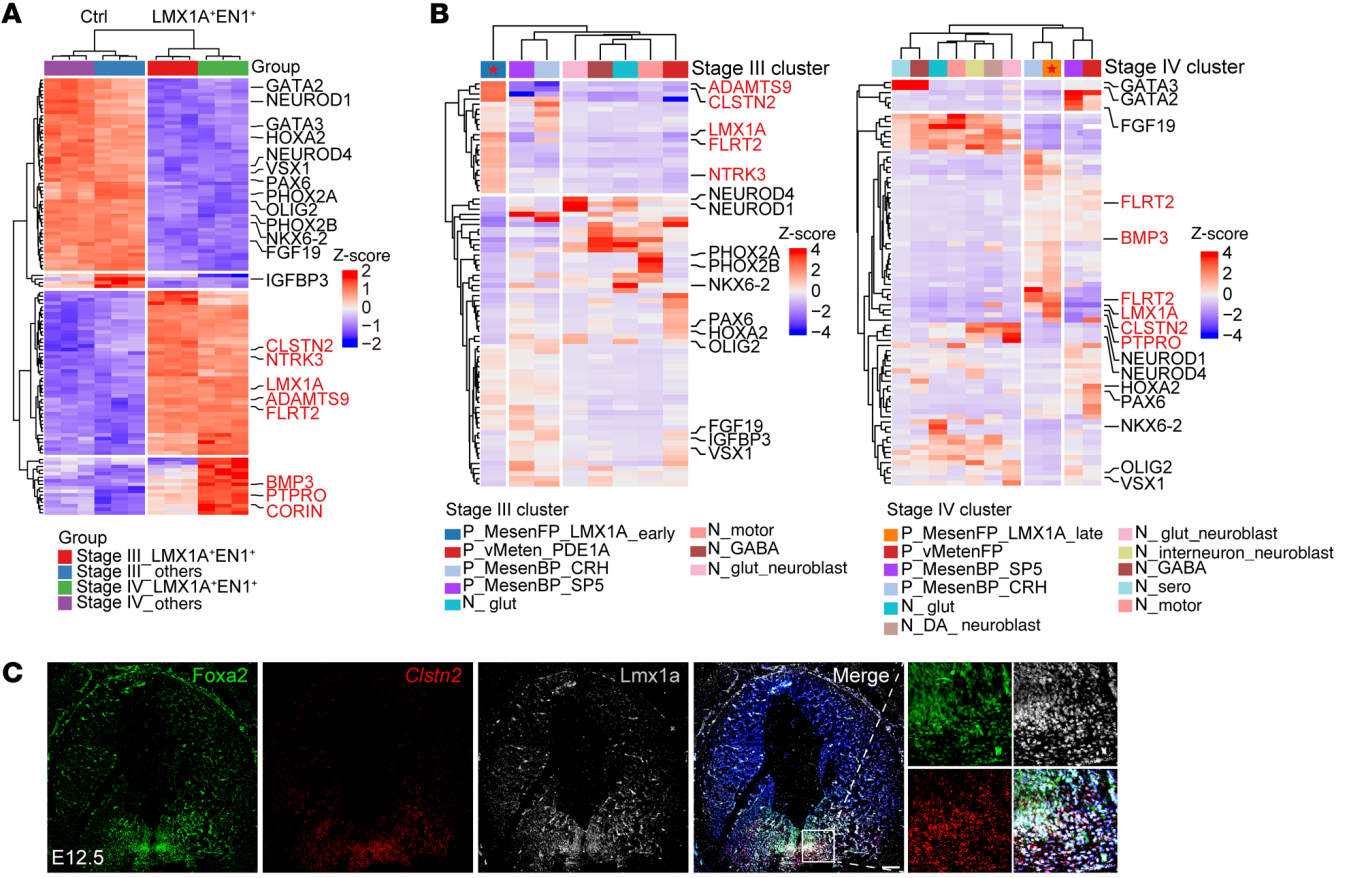
Identified surface markers are coexpressed with classical mDA progenitor markers in vitro and in developing mouse ventral midbrain. (**A**) Heatmap showing scaled expression of 4 groups of top 40 DEGs (stage III LMX1A^+^EN1^+^ and control; stage IV LMX1A^+^EN1^+^ and control). Control cells were collected from LMX1A^–^EN1^–^, LMX1A^+^EN1^–^, and LMX1A^–^EN1^+^. DEGs in LMX1A^+^EN1^+^ cells are shown in red, and DEGs in control cells are shown in black. (**B**) Heatmap showing scaled expression of same DEGs from **A** projected onto stage III (left) and stage IV (right) scRNA-Seq clusters. Marker genes are shown in the same color as in **A**. (**C**) RNA-FISH of *Clstn2* following IHC by colabeling Foxa2 and Lmx1a in E12.5 mouse mesencephalon. The zoomed views indicate magnified images of Foxa2^+^Lmx1a^+^*Clstn2*^+^ progenitors. Scale bars: 100 μm. Original magnification, ×20 (higher-magnification images).

To further validate the specific expression of CLSTN2 and PTPRO in the mDA progenitors, we differentiated LMX1A-tdT/EN1-mNeonG hPSCs and isolated LMX1A^+^EN1^+^ progenitors via FACS at stage III or IV (Supplemental Figure 3E). The remaining cells (LMX1A^–^EN1^–^, LMX1A^+^EN1^–^, and LMX1A^–^EN1^+^) were used as controls. Transcriptomic analysis showed that multiple genes, including *CLSTN2* and *PTPRO*, were highly expressed in LMX1A^+^EN1^+^ cells compared with control cells, confirming the specific expression of these 2 surface markers in mDA progenitors (Figure 6A, genes labeled in red, and Supplemental Figure 3F and Supplemental Table 3). We also identified *CORIN* as a DEG in LMX1A^+^EN1^+^ cells, which was previously reported as a marker of mesencephalic floor plate cells (37). Of note, many markers of non-mDA cells, such as the hindbrain marker *HOXA2* (38), the serotoninergic neuron and midbrain GABAergic neuron markers *GATA2* and *GATA3* (39), and the motor neuron progenitor markers *PHOX2A* and *PHOX2B* (40), were enriched in the control group (Figure 6A, genes labeled in black), indicating heterogeneous cell composition among non-LMX1A^+^EN1^+^ cells.

We next projected the DEGs identified from bulk RNA-Seq onto the scRNA-Seq data set (stage III and IV). DEGs enriched in LMX1A^+^EN1^+^ cells including *CLSTN2*, *PTPRO*, *LMX1A*, and *ADAMTS9*, were expressed specifically by single-cell mDA progenitor clusters at stage III or IV (Figure 6B, red star-labeled column), whereas DEGs enriched in the remaining cells (non-LMX1A^+^EN1^+^ cells) were distributed in non-mDA cell clusters in our scRNA data (Figure 6B). Note that only a few DEGs detected from bulk RNA-Seq analysis overlapped with DEGs from the scRNA-Seq data (Supplemental Figure 3, G–J). This may be explained by gene dropout in the scRNA-Seq (the gene was not detected in a given cell) or low-resolution of bulk RNA-Seq (genes expressed in a small number of cell populations were averaged). CLSTN2 is a single transmembrane protein belonging to the cadherin superfamily. PTPRO is a transmembrane protein belonging to the protein tyrosine phosphatase family. We verified the plasma membrane localization of both proteins (Supplemental Figure 3, K–M). FISH analysis for the RNA of *Clstn2* in the mouse embryo at E11.5 and E12.5, the major period of mDA neurogenesis, showed that *Clstn2* was specifically expressed in the ventral mesencephalon cells, which coexpressed the floor plate markers Foxa2 and Lmx1a (Figure 6C and Supplemental Figure 3N). Together, we have identified a set of markers, including CLSTN2, PTPRO, and LMX1A^+^EN1^+^, that were specifically expressed by mDA progenitors, where CLSTN2 and PTPRO represented early and late mDA progenitors, respectively.

### CLSTN2 and PTPRO are predictive of mDA neuron differentiation and can give rise to highly enriched mDA neurons after progenitor sorting and transplantation.

**Figure 7:**
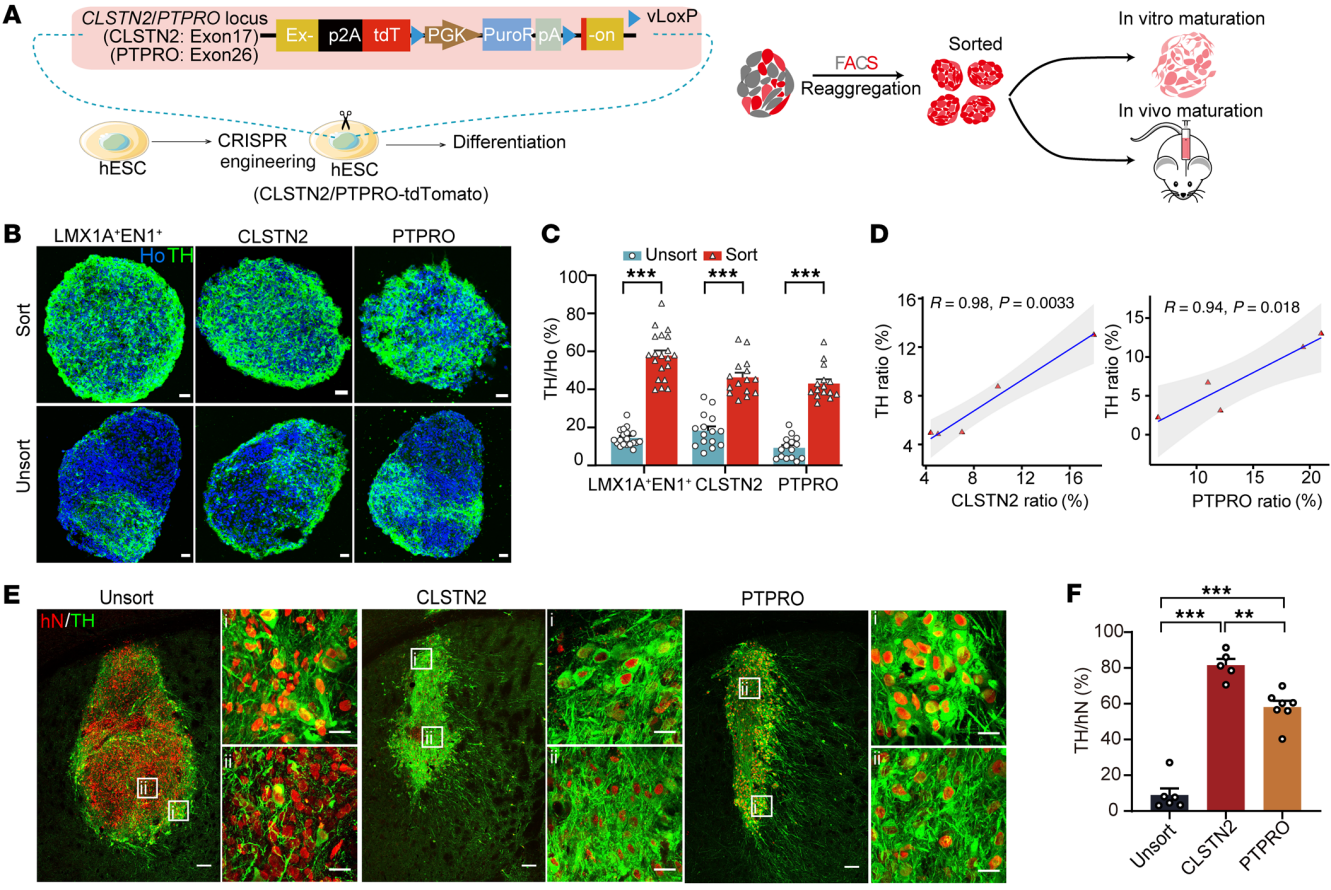
CLSTN2 and PTPRO are predictive of mDA neuron differentiation and can give rise to highly enriched mDA neurons after progenitor sorting and transplantation. (**A**) Diagram of surface marker reporter cell lines and experimental schematics for in vitro and in vivo maturation. (**B** and **C**) Neurospheres matured in vitro and (**B**) immunostained for TH and (**C**) statistical analysis (*n* = 3 batches with 5 neurospheres per batch). Scale bars: 25 μm. ***P < 0.001, by multiple unpaired *t* test with Holm-Šidák correction. (**D**) Correlation between the surface marker progenitor ratio and the TH^+^ neuron ratio. See Figure 9A for a diagram of the cell lines used. (**E**) Unsorted progenitor-, CLSTN2^+^ progenitor–, and PTPRO^+^ progenitor–derived grafts immunostained for human nuclei (hN) and TH. Scale bars: 100 μm and 20 μm (for the enlarged insets [i] and [ii], which represent the edge and center area of the graft, respectively). (**F**) Quantification of the TH^+^ neurons ratio in grafts. *n =* 6 (unsorted), *n* = 5 (CLSTN2), and *n* = 7 (PTPRO). **P < 0.01 and ***P < 0.001, by 1-way ANOVA followed by Tukey’s multiple-comparison test. Sort, sorted; unsort, unsorted.

We next tested whether CLSTN2 and PTPRO were sufficient to identify and enrich mDA progenitors. To address this, we created hPSC lines in which tdT was inserted into the C-terminal of either the *CLSTN2* or *PTPRO* gene (CLSTN2-tdT hPSCs and PTPRO-tdT hPSCs) (Figure 7A and Supplemental Figure 4A). We then isolated CLSTN2^+^ or PTPRO^+^ progenitors (tdT^+^) as well as LMX1A^+^EN1^+^ progenitors (tdT^+^mNeonGreen^+^) at designated stages of differentiation (stage III for CLSTN2^+^ progenitors, stage IV for PTPRO^+^ progenitors, and stages III and IV for LMX1A^+^EN1^+^ progenitors). A total of 10,000 cells were reaggregated into neurospheres and then matured in vitro to obtain terminal fates (Figure 7A and Supplemental Figure 4B). At early stages of maturation, the canonical mDA progenitor markers LMX1A and OTX2 were enriched in neurospheres from all marker-sorted groups compared with those derived from unsorted groups (Supplemental Figure 4, C–F). At the terminal stage, TH^+^ mDA neurons were highly enriched in neurospheres from the marker-sorted groups (LMX1A^+^EN1^+^, sorted: 57.7% ± 2.7%, unsorted: 14.5% ± 1.0%; CLSTN2, sorted: 46.3% ± 2.5%, unsorted: 18.3% ± 2.3%; PTPRO, sorted: 43.1% ± 2.3%, unsorted: 9.2% ± 1.5%; mean ± SEM, Figure 7, B and C). In addition, the percentage of CLSTN2^+^ or PTPRO^+^ cells at progenitor stages correlated well with the yield of mDA neurons in mature neurospheres (CLSTN2 group, *R* = 0.98, *P =* 0.0033; PTPRO group, *R* = 0.94, *P =* 0.018; Pearson’s correlation; Figure 7D), suggesting that these 2 markers are predictive of mDA neuron differentiation.

Next, we transplanted marker-sorted or unsorted progenitors into the dorsal striatum of a mouse model of PD (see Methods). Six months after transplantation, immunohistochemical analysis of the unsorted group revealed that TH^+^ mDA neurons were found primarily at the periphery of the grafts, as seen previously (22, 41, 42). In striking contrast, mDA neurons derived from CLSTN2^+^, PTPRO^+^, or LMX1A^+^EN1^+^ sorted groups were more evenly distributed throughout the grafts (Figure 7E and Supplemental Figure 4G). Importantly, the percentage of mDA neurons in the sorted grafts was dramatically elevated (PTPRO: 58.2% ± 3.5%; CLSTN2: 81.5% ± 3.5%; stage III LMX1A^+^EN1^+^: 48.3% ± 7.5%; stage IV LMX1A^+^EN1^+^: 32.4% ± 4.6%) compared with unsorted controls (9.0% ± 7.5%, mean ± SEM, Figure 7F and Supplemental Figure 4H). Most of the grafted cells in the marker-sorted groups expressed the floor plate marker FOXA2 (Supplemental Figure 5, A and B). Furthermore, most grafted mDA neurons coexpressed PITX3 and the dopamine transporter SLC6A3 (also known as DAT) (Supplemental Figure 5, C and D), suggesting they were mature mDA neurons. Further examination of mDA neuron subtypes showed that approximately 90% were A9-like (TH^+^GIRK2^+^), whereas approximately 10% were A10-like (TH^+^CB^+^) (Supplemental Figure 5, E and F). We found that CLSTN2 or PTPRO could also be used to enrich mDA neurons derived from hiPSC reporter lines (CLSTN2-XZ#2 TH^+^ neuron sorted: 50.2% ± 5.9%, unsorted: 9.0% ± 0.9%; PTPRO-ZYW#2 TH^+^ neuron sorted: 41.6% ± 3.8%, unsorted: 13.2% ± 2.8%; mean ± SEM, Supplemental Figure 5, G–N), or to enrich mDA neurons generated under feeder-free conditions (TH^+^ neuron sorted: 44.0% ± 4.2%, unsorted: 7.7% ± 0.8%; mean ± SEM, Supplemental Figure 5, O–Q).

Together, these results demonstrate that CLSTN2 and PTPRO, as well as the combination of LMX1A and EN1, were capable of predicting mDA neuron fate and enriching mDA progenitors, resulting in highly enriched mDA neurons after transplantation. Furthermore, enrichment of mDA neurons using these markers was robust and generalizable across different culture conditions (feeder and feeder-free) and for different hiPSC lines. This is critical if these procedures are to be used in personalized cell replacement therapies (43).

### CLSTN2- or PTPRO-enriched progenitors give rise to smaller grafts but denser dopaminergic innervations after transplantation.

**Figure 8:**
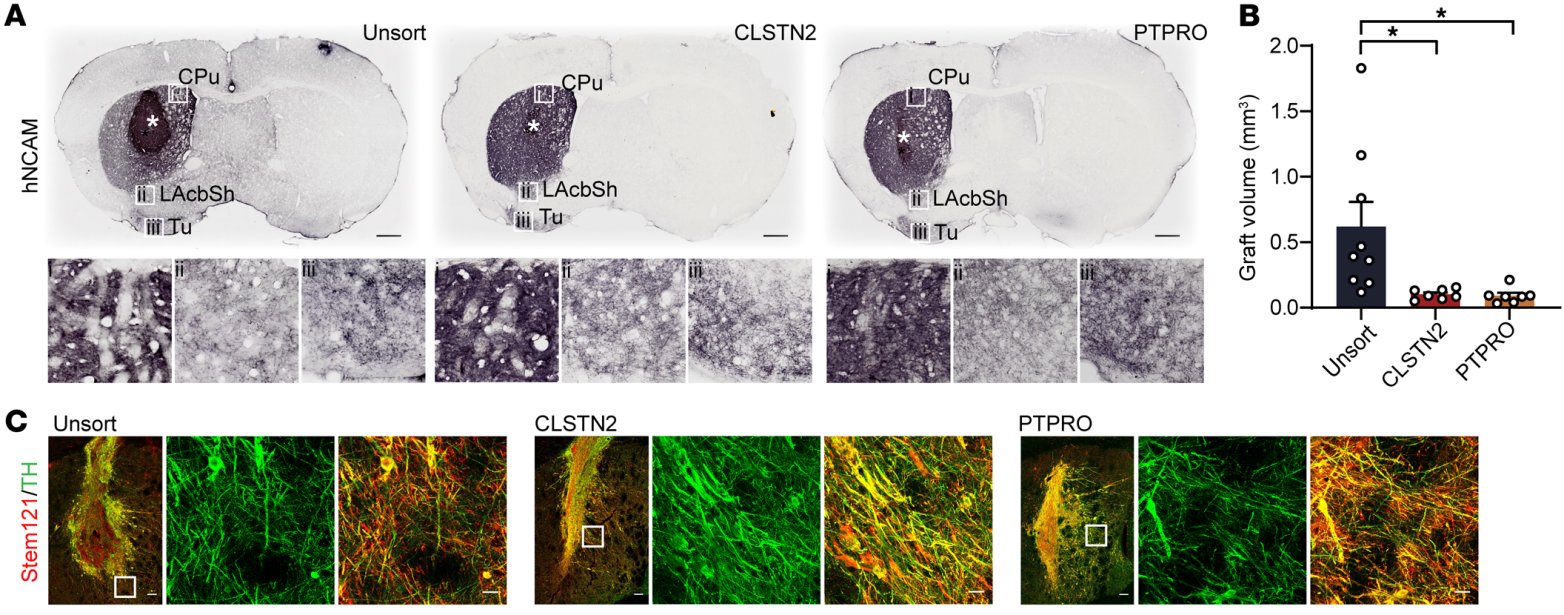
CLSTN2- or PTPRO-enriched progenitors reinnervate the host striatum and give rise to smaller grafts. (**A**) Immunostaining for hNCAM in grafted neurons showed hNCAM^+^ fiber distribution and extension into the dorsal striatum (caudate putamen [CPu], inset box i) and the ventral striatum (lateral nucleus accumbens shell [LAcbSh], inset box ii; olfactory tubercle [Tu], inset box iii). White asterisk indicates the graft site. Scale bars: 500 μm. (**B**) Graft volumes were estimated by hN staining at 6 months. *n =* 9 (unsorted), *n* = 7 (CLSTN2), *n* = 8 (PTPRO). *P < 0.05, by 1-way ANOVA followed by Tukey’s multiple-comparison test. (**C**) Grafts were colabeled for human-specific fiber STEM121 and TH. Scale bars: 100 μm and 20 μm (insets, representing zoomed views of extended graft fiber).

We next assessed graft innervation patterns by staining for human neural cell adhesion molecule (hNCAM). In all sorted or unsorted groups, we observed dense hNCAM^+^ fibers throughout the entire caudate putamen (CPu), the brain region targeted by endogenous DA neurons within the substantia nigra (Figure 8A and Supplemental Figure 6, A and B). We observed lower densities of hNCAM^+^ fibers in the lateral nucleus accumbens shell (LAcbSh) and the olfactory tubercle (Tu), target regions of endogenous mDA neurons within the ventral tegmental area (VTA) (Figure 8A and Supplemental Figure 6, A and B). Interestingly, marker-sorted grafts were much smaller than unsorted grafts, suggesting that sorting eliminated cells with a high proliferative potential (Figure 8B and Supplemental Figure 6C). Further examination showed that most of the STEM121^+^ human fibers derived from marker-sorted grafts expressed TH (Figure 8C and Supplemental Figure 6D), demonstrating a dopaminergic identity. Human-specific synaptophysin puncta were distributed along TH^+^ fiber in the CPu in marker-sorted grafts, suggesting that grafted mDA neurons form synaptic connections with host neurons (Supplemental Figure 6E).

**Figure 9:**
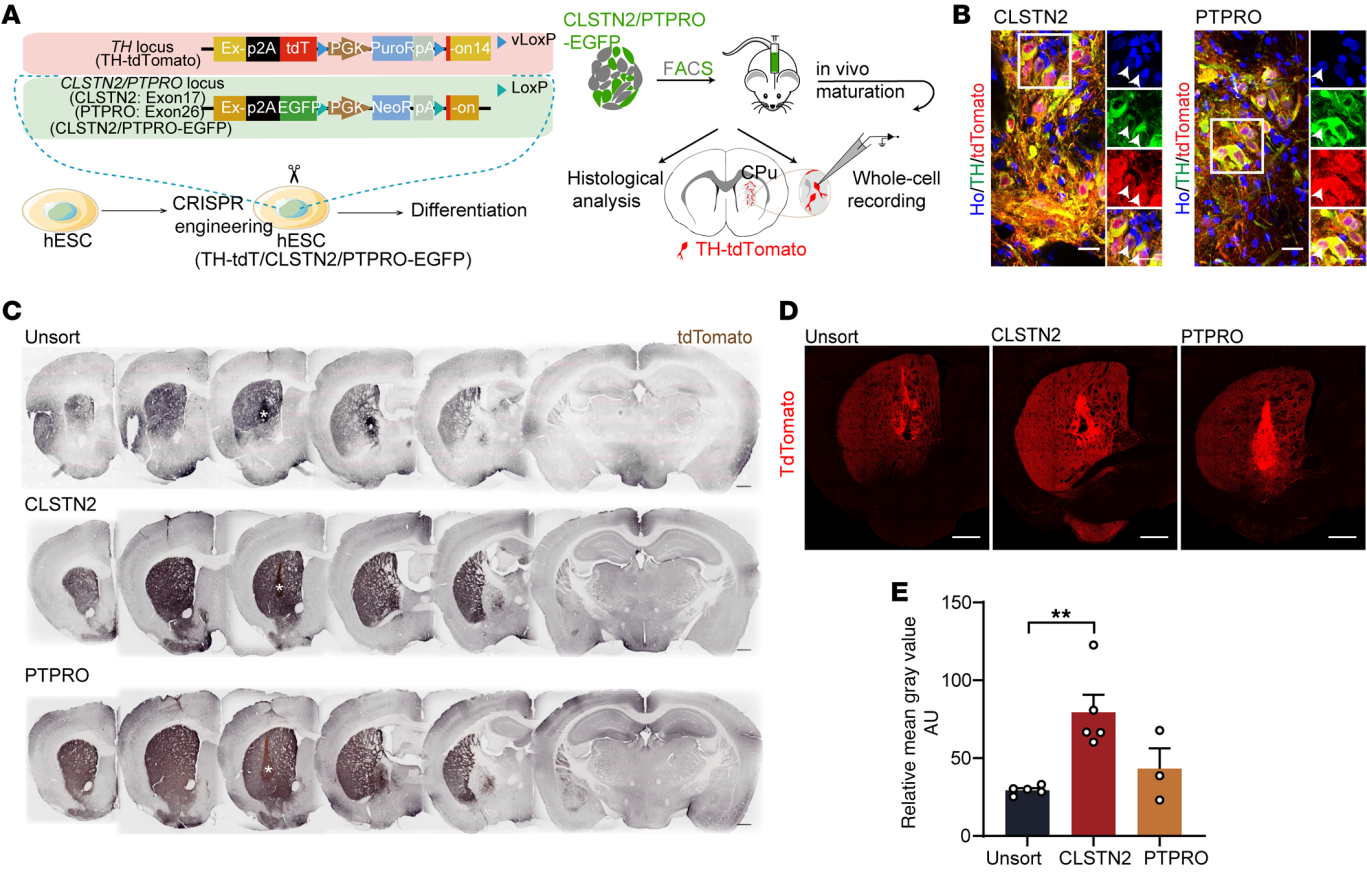
CLSTN2- or PTPRO-enriched progenitors give rise to denser DA innervations after transplantation. (**A**) Schematics for TH-specific histological evaluation and electrophysiological recording in surface marker–derived grafts. (**B**) Immunostaining for tdT in TH^+^ neurons in CLSTN2- and PTPRO-derived grafts. Boxed areas are magnified on the right. White arrows indicate neurons coexpressing tdT and TH. Scale bars: 20 μm. Original magnification, ×60 (enlarged insets). (**C**) Serial coronal sections of grafts immunostained for tdT. White asterisk indicates the graft site. Scale bars: 500 μm. (**D**) Typical IHC images with tdT labeling (representing TH) in grafts. Scale bars: 500 μm. (**E**) Quantification of the mean gray value of tdT pixels from 4 random areas within the host striatum (see Supplemental Methods for details). **P < 0.01, by 1-way ANOVA followed by Tukey’s multiple-comparison test.

To specifically elucidate the axonal innervation by grafted mDA neurons, we constructed cell lines that expressed tdT via TH and EGFP via the *CLSTN2* or *PTPRO* marker genes (TH-tdT/CLSTN2-EGFP hPSCs, and TH-tdT/PTPRO-EGFP hPSCs). TH-tdT hPSCs served as unsorted controls (Figure 9A and Supplemental Figure 7A). EGFP^+^ cells were readily detected at progenitor stages in vitro (Supplemental Figure 7B). We then transplanted surface marker–sorted or unsorted progenitors derived from these hPSC reporter lines into the striatum of PD mice. Five months after transplantation, tdT was expressed exclusively in TH^+^ mDA neurons in the grafts (Figure 9B). Consistent with the innervation patterns of hNCAM^+^ fibers, tdT^+^ human mDA fibers from marker-sorted or unsorted grafts were distributed throughout the CPu (Figure 9C). Importantly, tdT^+^ human mDA fibers from marker-sorted grafts were denser than those from unsorted grafts, suggesting that surface marker–sorted grafts provide stronger dopaminergic innervation (Figure 9, D and E).

### CLSTN2- or PTPRO-enriched progenitors integrate into host circuits and exhibit higher therapeutic potency.

**Figure 10:**
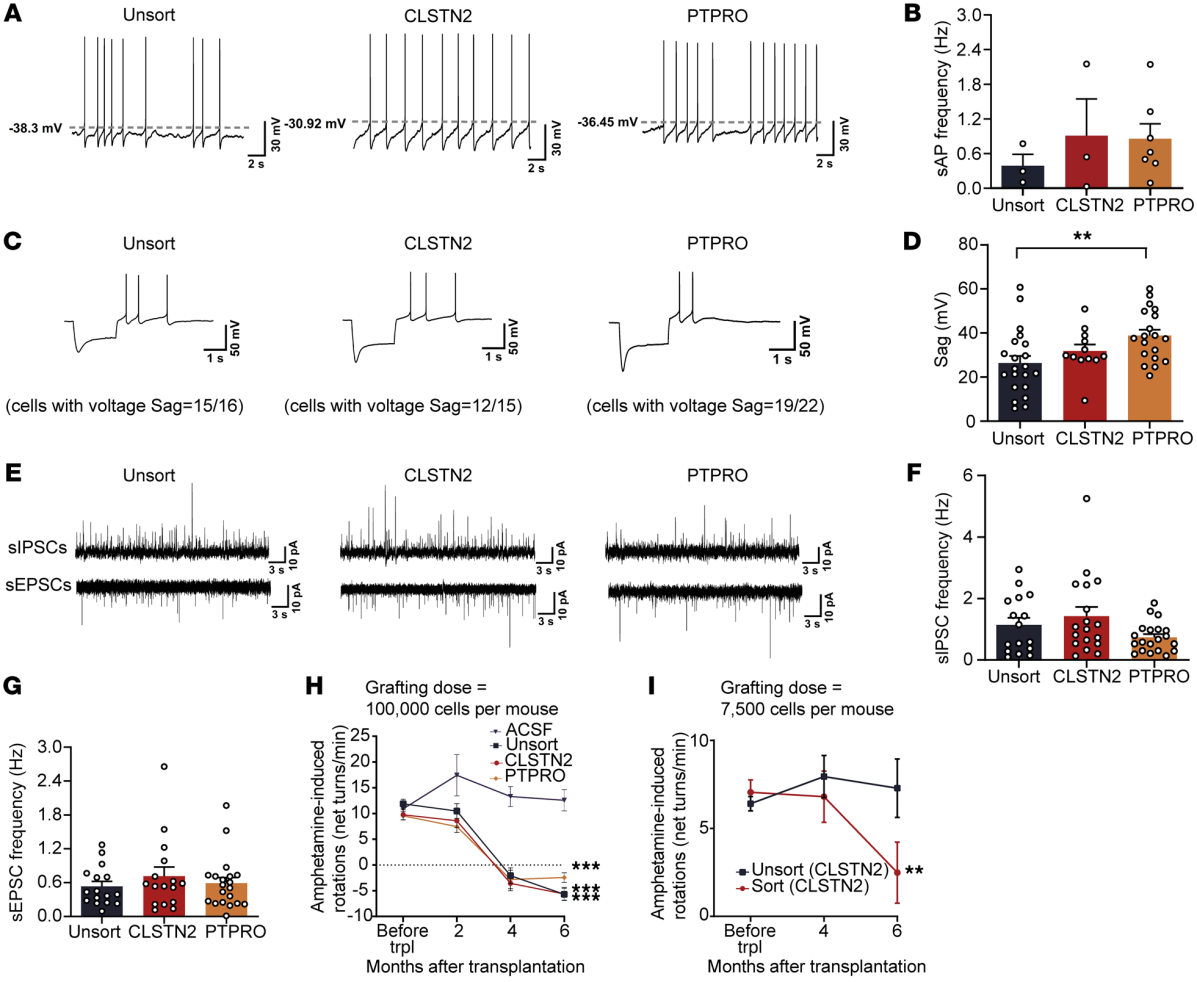
CLSTN2- or PTPRO-enriched progenitors integrate into host circuits and exhibit a higher therapeutic potency. (**A**–**D**) Typical traces of whole-cell patch-clamp recording of sAPs (**A**) and sAP frequency (**B**), hyperpolarizing current injection showing voltage sag (**C**), and voltage sag measurements (**D**) from grafted mDA neurons 5 months after transplantation. Recorded cell numbers: *n =* 24 (unsorted), *n* = 15 (CLSTN2), *n* = 22 (PTPRO). **P < 0.01, by 1-way ANOVA followed by Tukey’s multiple-comparison test. (**E**) Typical traces of sIPSCs (top) and sEPSCs (bottom) in grafted human mDA neurons 5 months after transplantation. (**F** and **G**) Frequencies of sIPSCs (**F**) and sEPSCs (**G**). Number of mice: *n =* 4 (unsorted), *n* = 3 (CLSTN2), *n* = 4 (PTPRO). Recorded cell numbers for sEPSCs: *n =* 16 (unsorted), *n* = 16 (CLSTN2), *n* = 20 (PTPRO). Recorded cell numbers for sIPSCs: *n =* 16 (unsorted), *n* = 18 (CLSTN2), *n* = 20 (PTPRO). (**H** and **I**) Amphetamine-induced rotation behavior changes in PD mice over a 6-month post-transplantation period. The grafting dose per mouse was 100,000 cells (**H**). *n =* 5 (aCSF), *n* = 9 (unsorted), *n* = 11 (CLSTN2), *n* = 9 (PTPRO). (**I**) The grafting dose per mouse was 7500 cells. The H9-CLSTN2-P2A-tdT cell line was used. *n =* 4 (unsorted), *n* = 3 (sorted). The tdT ratio for the unsorted group was approximately 29%. **P < 0.01 and ***P < 0.001, by 2-way ANOVA with Dunnett’s multiple-comparison test, compared with the ACSF group (**H**) or with the unsorted group (**I**). trpl, transplantation.

Harnessing genetic reporters (Figure 9A), we next examined the electrophysiological properties of grafted mDA neurons. Whole-cell patch-clamp recordings revealed that human mDA neurons (tdT^+^) within sorted or unsorted grafts displayed similar spontaneous action potential (sAPs) and current-induced APs 5 months after transplantation, suggesting that they were functionally mature (Figure 10A and Supplemental Figure 7, C and D). The resting membrane potential (RMP) and AP threshold were comparable between sorted and unsorted groups (Supplemental Figure 7, E and F). For all groups, sAPs regularly discharged with low spiking rates (unsorted, 0.39 Hz; CLSTN2, 0.91 Hz; PTPRO, 0.86 Hz; Figure 10, A and B) and afterhyperpolarization (AHP) was prominent, features consistent with those of endogenous SNc (A9) mDA neurons (22) (Supplemental Figure 7G). Further, most grafted mDA neurons in all groups exhibited sag potentials in response to injection of a hyperpolarizing current, a typical characteristic of A9 mDA neurons (44–46) (Figure 10C). Notably, grafted mDA neurons in the CLSTN2- or PTPRO-sorted group generally displayed comparable depolarizing voltage in AHP (unsorted, –6.9 mV; CLSTN2, –9.6 mV; PTPRO, –7.7 mV; Supplemental Figure 7G) and amplitude of sag compared with the unsorted group (unsorted, 26 mV; CLSTN2, 32 mV; PTPRO, 39 mV; Figure 10, C and D). This suggested mature hyperpolarization-activated cyclic nucleotide–gated (HCN-gated) channels and T-type calcium channel function (47, 48). Moreover, grafted mDA neurons show a depolarization block after reaching maximal firing frequency in response to the injection of increasing ramp currents (Supplemental Figure 7, H and I). These results indicate that both sorted and unsorted mDA neurons became functionally mature 5 months after transplantation and that most had electrophysiological characteristics of A9 mDA neurons.

We previously reported that functional inputs to grafted mDA neurons are established 3–6 months after transplantation (22). Here, electrophysiological analysis of grafted mDA neurons showed that spontaneous excitatory and inhibitory postsynaptic currents (sEPSCs and sIPSCs, respectively) were readily detected in grafted mDA neurons 5 months after transplantation (Figure 10E). The mean amplitude and frequency of sIPSCs and sEPSCs were comparable between the sorted and unsorted groups (Figure 10, F and G, and Supplemental Figure 7, J and K). These results demonstrate that both surface marker–sorted and unsorted mDA neurons integrated into host circuits presynaptically and received functional inputs.

Next, we assessed the functional effect of transplanted cells by amphetamine-induced rotation before and every 2 months after grafting. PD mice that received LMX1A^+^EN1^+^, CLSTN2-, or PTPRO-sorted progenitors or unsorted progenitors gradually recovered over time from the amphetamine-induced rotation behavior (Figure 10H and Supplemental Figure 7L), whereas those that received only artificial cerebrospinal fluid (aCSF) did not (Figure 10H). On the basis of these results and the fact that marker-sorted cells were highly enriched for mDA progenitors, we reasoned that we could transplant fewer cells and still see a functional recovery of PD mice. We therefore reduced the number of transplanted cells from 100,000 to 7500 (less than 10% of the original transplantation dose) per mouse. Six months after transplantation, we observed recovery from amphetamine-induced rotation behavior in mice that received marker-sorted progenitors, but not in those that received unsorted progenitors, indicating that marker-sorted mDA progenitors had higher therapeutic potency (Figure 10I).

### Grafts from CLSTN2- or PTPRO-enriched progenitors have consistent cellular composition, with mDA neurons enriched and most off-target neuron types depleted.

**Figure 11:**
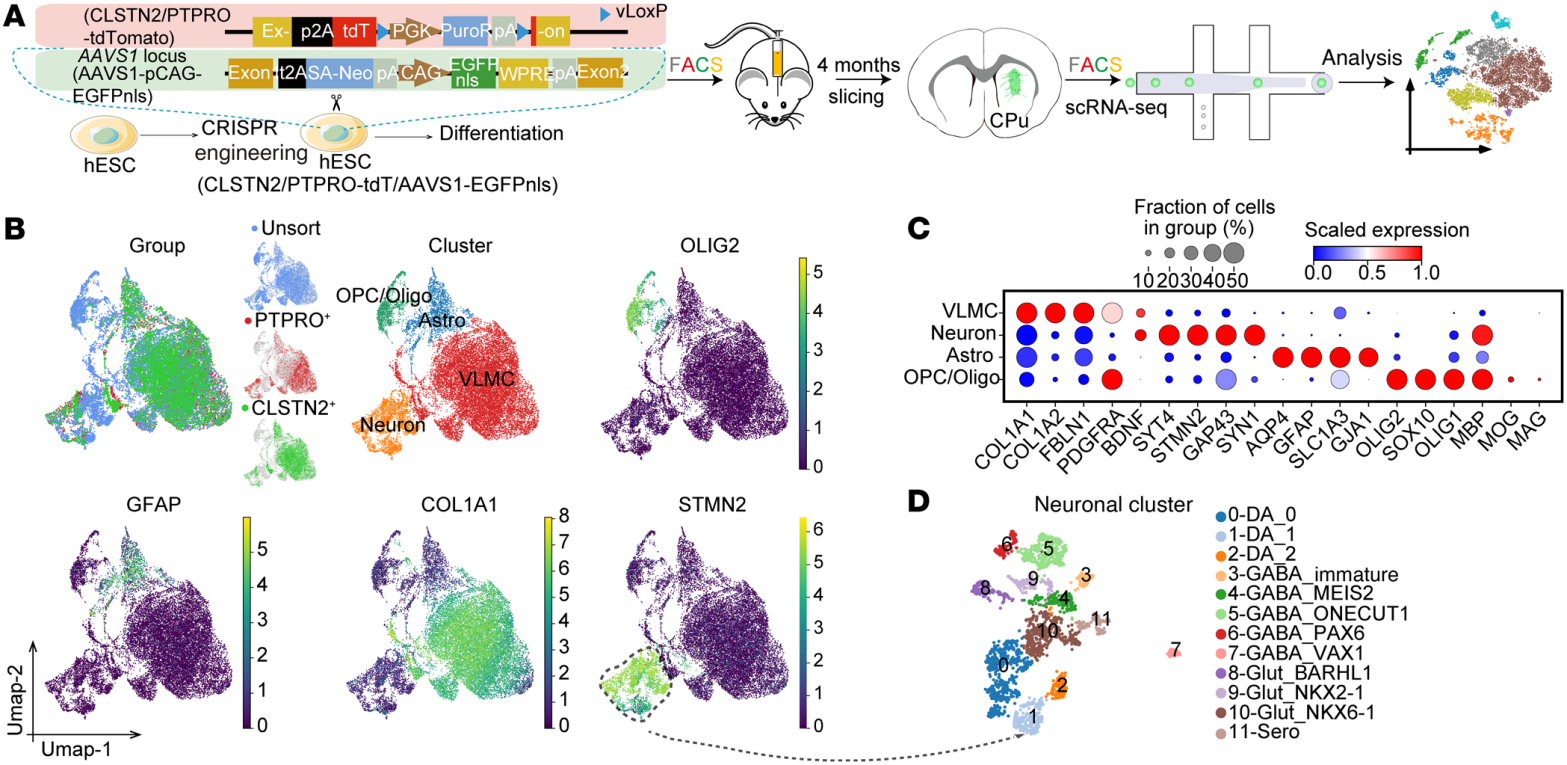
scRNA-Seq reveals the cellular composition of grafts. (**A**) Schematics for scRNA-Seq of grafts. (**B**) Clustering recovered 4 major cell types in grafts and their corresponding typical gene expression. (**C**) Dot plot showing markers of the cell types in grafts. (**D**) Further clustering of neurons.

Having established key functional differences between sorted and unsorted mDA grafts, we next sought to define the cellular composition of these grafts by scRNA-Seq. To remove the contamination of host (mouse) cells in the cell suspensions when dissecting graft tissue, we knocked a nuclear EGFP-expressing cassette (EGFP-nucleoplasmin, EGFPnls) into the *AAVS1* locus of surface marker reporter hPSC lines (CLSTN2-tdT hPSCs or PTPRO-tdT hPSCs) to label all of the human cells (Figure 11A and Supplemental Figure 8A). We transplanted surface marker–sorted or unsorted mDA progenitors derived from these hPSC lines into the striatum of PD mice. Four months after transplantation, we dissected grafts and prepared single-cell suspensions. Grafted human cells (EGFP^+^) were then isolated via FACS and subjected to scRNA-Seq analysis (Figure 11A). Clustering analysis revealed 4 major cell types, including oligodendrocyte progenitor cells and oligodendrocytes (the OPC/oligo cluster), astrocytes (astro cluster), neurons (neuron cluster), and previously reported vascular leptomeningeal-like cells (VLMC cluster) (49) (Figure 11, B and C).

Histological analysis showed that OPC/oligo cells, but not astrocytes, were largely absent from sorted grafts compared with unsorted grafts (Supplemental Figure 8, B–E). As it was difficult to accurately identify the number of VLMCs, we calculated the coverage of VLMCs in the grafts and found that coverage varied across groups (Supplemental Figure 8, F and G). VLMCs could be further clustered into 6 subtypes, some of which expressed neurotrophic factors or neurotrophic factor–related proteins, such as *BDNF*, *BMP7*, and *IGFBP2* (Supplemental Figure 8, H and I).

**Figure 12:**
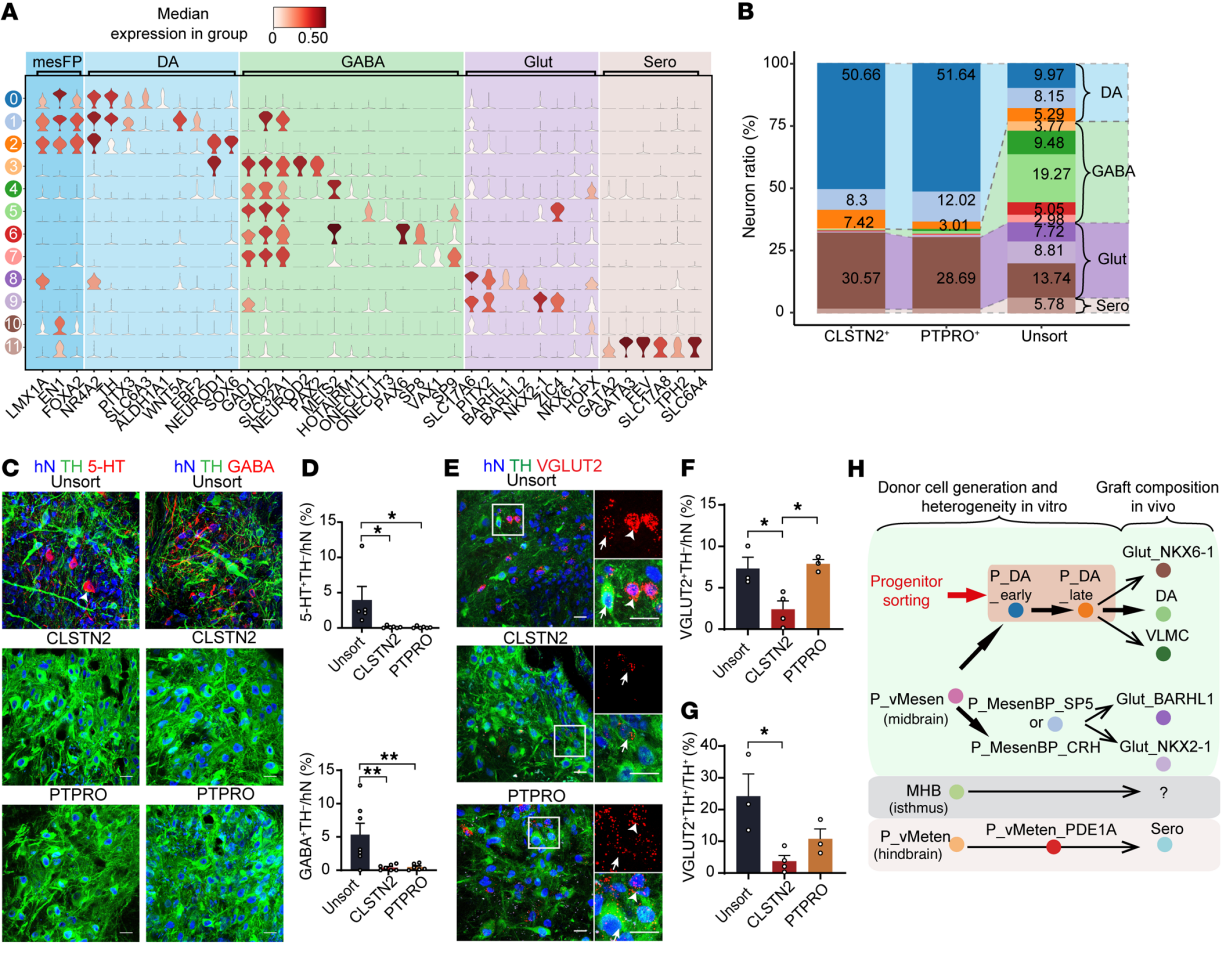
Grafts from CLSTN2- or PTPRO-enriched progenitors contain enriched mDA neurons with most off-target neuron types depleted. (**A**) Violin plot of representative markers for all graft neuronal clusters. (**B**) Neuronal subtype ratio for each graft group (unsorted, CLSTN2, and PTPRO). (**C** and **D**) Typical images of grafts immunostained for 5-HT (**C**, left) or GABA (**C**, right) and the mDA marker TH. Quantification of 5-HT^+^ (**D**, top) and GABA^+^ neuron ratios (**D**, bottom) in grafts for each group. *P < 0.05 and **P < 0.01, by 1-way ANOVA followed by Tukey’s multiple-comparison test. (**E**–**G**) Representative images of grafts immunostained for VGLUT2 (RNAScope) and TH (**E**). Arrowheads and arrows indicate VGLUT2^+^TH^–^ neurons and TH^+^ neurons with weak VGLUT2 expression, respectively. Quantification of VGLUT2^+^TH^–^/hN ratio (**F**) and the VGLUT2^+^TH^+^/TH^+^ ratio (**G**). Scale bars: 20 μm. *P < 0.05, by 1-way ANOVA followed by Tukey’s multiple-comparison test. (**H**) Proposed model of how heterogenous donor cells generated in vitro result in grafts with diverse neuronal composition in vivo. Graft outcomes can be improved and predicted after mDA progenitor sorting via specific markers.

Focusing on neurons, we further identified 12 neuronal clusters from 1459 neurons (Supplemental Methods). Each cluster could be distinguished by representative markers (Figure 11D and Figure 12A). Cell-type replicability assessment showed that neurons in the grafts were transcriptionally similar to those in the developing midbrain (Supplemental Figure 8J). Three mDA neuron subtypes expressing *TH* and *PITX3* were detected (DA_0, DA_1, and DA_2). The most abundant of these clusters, DA_0, expressed the dopamine transporter *SLC6A3* (*DAT*) and the A9 (SNc) mDA neuron marker *ALDH1A1* (50), indicative of mature A9 mDA neurons. The DA_1 cluster expressed *GAD2* and *SLC32A1*, suggesting an mDA neuron subtype that also releases GABA (Figure 12A). The DA_2 cluster expressed *NEUROD1* and *TH*, albeit weakly, indicating that the neurons in this cluster were in the early mature stage (Figure 12A). In addition to these mDA neurons, we detected 5 subtypes of GABAergic neurons, 3 subtypes of glutamatergic neurons, and 1 type of serotoninergic neurons, indicating the presence of various types of neurons in the graft (Figure 11D and Figure 12A).

We then calculated the percentage of each neuronal subtype in each group (Figure 12B). Similar to our histological results, mDA neurons were enriched in CLSTN2 and PTPRO sorted grafts (Figure 7F and Figure 12B). In contrast, serotoninergic neurons, the potential source of graft-induced dyskinesia, and GABAergic neurons were largely absent from the CLSTN2- and PTPRO-sorted grafts (Figure 12B). Further histological examination confirmed the absence of both serotoninergic and GABAergic neurons in surface marker–sorted grafts as well as in stage III or IV LMX1A^+^EN1^+^ sorted grafts (Figure 12, C and D, and Supplemental Figure 8K).

Marker sorting reduced the number of glutamatergic neuronal subtypes from three (Glut_BARHL1^+^ cluster, Glut_NKX2-1^+^ cluster, and Glut_NKX6-1^+^ cluster in unsorted group) to one (Glut_NKX6-1^+^ cluster in CLSTN2- or PTPRO-sorted group), whereas the overall percentage of glutamatergic neurons was similar between sorted and unsorted grafts. IHC and RNA-FISH confirmed the presence of human glutamatergic neurons (*VGLUT2*^+^TH^–^; Figure 12E, arrowheads) in all 3 groups (unsorted, 7.3%; CLSTN2, 2.4%; PTPRO, 7.9%) (Figure 12, E and F). A small portion of mDA neurons also weakly expressed *VGLUT2*, suggesting an mDA neuron subtype releasing glutamate (*VGLUT2*^+^TH^+^/TH^+^ ratio, unsorted: 24.2%; CLSTN2: 3.7%, PTPRO: 11.8%; Figure 12E, arrows, and Figure 12G). The expression of *VGLUT2* (*SLC17A6*) in mDA neurons was also supported by scRNA-Seq data (Figure 12A). Of note, the grafts from different surface marker–sorted groups had strikingly similar neuronal compositions, with approximately 70% mDA neurons and approximately 30% NKX6-1^+^ glutamatergic neurons (Figure 12B), suggesting the potential for stable graft outcomes upon transplantation of marker-sorted mDA progenitors.

Altogether, we have resolved the cellular composition of grafts from both sorted and unsorted groups by scRNA-Seq and histological analysis. Our results revealed a remarkably consistent and homogeneous cellular composition of grafts generated by novel surface markers representing early (CLSTN2 at stage III) or late (PTPRO at stage IV) fate-committed mDA progenitors, with striking mDA neuron enrichment and elimination of unwanted neuron subtypes.

## Discussion

hPSCs offer a multitude of exciting opportunities for cell replacement therapies for devastating human diseases like PD. But for this approach to be broadly useful, safe, and efficacious, it is axiomatic that we must transplant as many of the right cells and as few of the wrong cells as possible and make sure that the cell products are uniform from batch to batch. The solution to these problems requires an understanding of developmental trajectories of neuronal differentiation and a method that can accurately separate target cells and eliminate unwanted cells. Here, using scRNA-Seq, we comprehensively characterized the cells that emerged during mDA neuron differentiation from hPSCs. Surprisingly, we found that our in vitro mDA neuron differentiation accurately recapitulated the development of multiple but adjacent fetal brain regions in vivo including the ventral midbrain (the origin of mDA lineage), the isthmus, and the ventral hindbrain. These results not only demonstrate that the mDA progenitors we generated in vitro were equivalent in developmental process and molecular characteristics to their in vivo counterparts, but also explain the cause and origin of unwanted cells among the donor cells. We speculate that these unwanted cells generated in vitro may be inevitable, which is the cost of hPSC-based neuronal differentiation to stimulate in vivo neurodevelopment. However, further efforts could be made to optimize the differentiation protocol to increase the proportion of ventral midbrain progenitors. More important, by analyzing the molecular dynamics of mDA lineage specification, we identified specific surface markers for mDA early or late progenitors, which were predictive of mDA neuron fate and could be used to purify mDA progenitors. We provide evidence in a PD mouse model that these purified mDA progenitors had greater therapeutic potency. By scRNA-Seq and histological analysis, we further demonstrated that these markers could give rise to stable grafts with predictable outcomes, with grafts composed of highly enriched mDA neurons and depleted off-target neurons. We believe that similar strategies could be applied to generate safer, quality-controllable cell products for other hPSC-based therapeutics.

The low mDA neuron ratio in grafts has been a major obstacle to PD cell therapy (10, 16, 18, 26, 51, 52). One way to address this issue is to identify markers of mDA progenitor for purification and enrichment. Several mDA progenitor markers, such as the transcription factor LMX1A (53), and surface markers (54, 55) have been used to enrich cells before transplantation. These markers were identified using traditional knowledge of mouse midbrain development or by their coexpression with classical ventral midbrain markers such as LMX1A or FOXA2 (53–55). However, these and several other classical ventral midbrain markers are also coexpressed in progenitors in other brain regions. For instance, LMX1A is expressed in progenitors of the subthalamic nucleus in diencephalon (26, 56). CORIN, an early surface marker of the floor plate that has been used to enrich putative mDA progenitors (54), is expressed not only in the ventral midbrain but in the ventral metencephalon and spinal cord (37). Indeed, it has been reported that the expression of *FOXA2*, *LMX1A*, and *CORIN* in donor cells does not have a positive correlation with human mDA neuron yields after transplantation in PD cell therapy (16), indicating that theses marker do not exclusively represent mDA progenitors. The mDA neuron ratio in the sorted grafts obtained using markers identified by these strategies was often not dramatically elevated (53–55).

Our time-course scRNA-Seq data sets and reconstructed trajectory of mDA neuron differentiation allowed us to unbiasedly identify CLSTN2 and PTPRO as surface markers that were specifically expressed in early and late mDA progenitors. However, these 2 genes have not been well studied in the literature. To our knowledge, there are currently no commercially available antibodies against CLSTN2 and PTPRO for live-cell sorting. Thus, we generated CLSTN2-tdTomato and PTPRO-tdTomato knock-in hPSC lines to identify CLSTN2- or PTPRO-expressing progenitor cells. Using these reporter cell lines, we demonstrated that purified progenitor cells expressing CLSTN2 or PTPRO gave rise to highly enriched mDA neurons (60%–80%) after transplantation (Figure 7, E and F), compared with unsorted grafts (~10% on average) and results from 2 recent preclinical studies (~10% TH^+^ cells) (18, 57). Another advantage of these surface markers was the low cell dose required for correcting motor deficits in the PD mouse model. In previous studies, 100,000 to 400,000 unsorted or marker-sorted cells per mouse or rat were unilaterally transplanted (5, 6, 16, 54), whereas our cell-dose experiment demonstrated rotation behavior recovery of PD mice 6 months after transplanting only 7500 sorted cells (Figure 10I), suggesting a high therapeutic potency. Most strikingly, we found that different surface marker–sorted grafts had a surprisingly consistent cellular composition as revealed by our scRNA-Seq analysis and histological validation, suggesting stable grafts and predictable graft outcomes (Figure 11, Figure 12, and Supplemental Figure 8). The ability to produce highly purified donor cells with predictable therapeutic outcomes represents a significant step forward, toward safer, more effective stem cell therapies. Further efforts are needed to generate the antibodies against CLSTN2 or PTPRO to facilitate the direct sorting of mDA progenitors in cell replacement therapy for PD in the clinic.

Since fetal tissues or hPSC-derived progenitors used for PD cell therapy are often heterogeneous and multipotent, it is important to identify the cell types, especially neurons, that have been generated in the graft following transplantation (10, 58). A recent study used scRNA-Seq to dissect the cellular composition of fetal and hPSC-derived grafts in a rat PD model (49). However, potentially because of the limited number of recovered neurons from the grafts, no neuronal heterogeneity was observed in that study. Taking advantage of the genetically labeled hPSC lines and scRNA-Seq, we observed a great deal of cellular heterogeneity in unsorted grafts, including 3 types of mDA neurons, as well as GABAergic, glutamatergic, and serotonergic neurons. These neuronal cell types were further verified by histological analysis that revealed results consistent with previous reports showing GABAergic and serotonergic neurons in the grafts (8–10). To our knowledge, glutamatergic neurons have not been previously reported in mDA grafts, potentially because of the lack of available antibodies or because different vesicular glutamate transporters are expressed in different types of glutamatergic neurons (VGLUT1, VGLUT2, and VGLUT3). Here, for the first time to our knowledge, we identified *VGLUT2*^+^ glutamatergic neurons in the unsorted grafts, counterparts of which reside in the developing mouse midbrain (27, 59). Although we applied scRNA-Seq to uncover the neuronal heterogeneity of the grafts, it is worth noting that the low recovery rate of neurons in the adult brain in scRNA-Seq experiments is a common technical challenge, as enzymatic and mechanical forces tend to destroy neurons containing extensive projections during the preparation of the single-cell suspension (49, 60, 61). Thus, more non-neurons (e.g., VLMC type) rather than neurons in the human grafts will be recovered and analyzed in the scRNA-Seq experiments. That is why we only obtained a minority of DA neurons in the grafts in our scRNA-Seq analysis. It is important to use alternative methods to validate the qualitative and quantitative results from scRNA-Seq data sets (49). In fact, our histological analysis clearly showed that the proportion of DA neurons in the sorted grafts was dramatically elevated (60%~80% of total human cells in the grafts) compared with that in the unsorted groups (~10%).

Our single-cell transcriptional atlas for in vitro mDA cultures and mDA grafts in vivo provide clues for understanding the origin of off-target cells in transplants in PD cell therapy (Figure 12H). Strikingly, at the early stage of mDA neuron differentiation, regional progenitors, including ventral midbrain, isthmus, and ventral hindbrain progenitors, already emerged and populated, and the diversity of progenitor cell types increased with differentiation. We speculate that the non-mDA progenitors, especially those that emerged in the middle or late stages (the time points we used for cell transplantation), are the major source of off-target neurons observed in the grafts after transplantation. For instance, at the middle and late stages of differentiation, we detected 2 midbrain basal plate progenitor clusters — P_MesenBP_SP5 and P_MesenBP_CRH — that expressed the basal plate marker *PITX2* and the basal/floor plate marker *FOXA2* (Figure 1C and Supplemental Figure 1B) (26, 59). A lineage-tracing study in developing mouse midbrain showed that Pitx2-derived progenitors could give rise to glutamatergic neurons (59). Interestingly, in the graft, we identified 2 glutamatergic clusters, Glut_NKX2-1 and Glut_BARHL1, which also specifically expressed *PITX2* (Figure 12A). The sharing of a key regional marker between these 2 basal plate progenitors and glutamatergic neurons suggests that P_MesenBP_SP5 and P_MesenBP_CRH progenitors may potentially give rise to Glut_NKX2-1 and Glut_BARHL1 after transplantation (Figure 12H). Interestingly, the third type of glutamatergic neuron detected in grafts, Glut_NKX6-1, expressed classical markers of mesencephalic floor plate, including *LMX1A*, *EN1*, and *FOXA2* (Figure 12A), indicating that NKX6-1^+^ glutamatergic neuron may have the same origin as mDA neurons, that is, the mesencephalic floor plate. This may explain why only NKX6-1^+^ glutamatergic neurons, but not NKX2-1^+^ or BARHL1^+^ glutamatergic neurons, were present in both CLSTN2-sorted and PTPRO-sorted grafts.

We also identified metencephalic progenitors (P_vMeten_PDE1A) at the mid-to-late stages of differentiation (stages III–V). These cells expressed *FOXA2* and *EN1* but not *LMX1A* or *OTX2* (Figure 1C and Supplemental Figure 1A), an expression signature pattern reminiscent of endogenous ventral hindbrain progenitors in rhombomere 1 (r1), which give rise to neuronal cells including serotonin neurons in the dorsal raphe of the hindbrain (28, 62, 63). Intriguingly, serotonin neurons detected in the unsorted grafts expressed high levels of *EN1* (Figure 12A), indicating that these serotonin neurons originated from r1 rather than more posterior regions, such as r2 or r3, which normally express HOXA2, but not EN1 (64). Thus, P_vMeten_PDE1A cells were the potential source of serotonin neurons in the unsorted grafts (Figure 12H). Further lineage-tracing studies are required to determine the lineage relationships between in vitro–generated progenitor cell types and mature neuron types in the grafts.

We identified a non-neural cell type in mDA grafts, namely VLMCs, whose presence and distribution in mDA grafts has recently been reported (49). Interestingly, VLMCs arise only in hPSC-derived mDA grafts, but not in grafts derived from fetal ventral midbrain tissue (49), suggesting that they may be products of in vitro differentiation. The function of VLMCs in PD cell therapy is unknown, however, our scRNA-Seq data revealed that some VLMCs expressed neurotrophic factors, suggesting a potential supportive role (Supplemental Figure 8H). It is worth noting that VLMCs were detected in the grafts from all of the 4 sorted groups (CLSTN2, PTPRO, stage III LMX1A^+^EN1^+^, and stage IV LMX1A^+^EN1^+^), in which highly purified mDA progenitors were transplanted. These results suggest that VLMCs may potentially originate from mDA progenitors (Figure 12H). On the basis of these findings, we hypothesize that hPSC-derived mDA progenitors are multipotent and could thus could generate mDA neurons, NKX6-1^+^ glutamatergic neurons, and VLMCs after transplantation (Figure 12H). Further studies are required to determine the cellular lineages and functions of VLMCs and NKX6-1^+^ glutamatergic neurons in PD cell replacement therapies. Altogether, we assume that some of the off-target cells observed in the unsorted grafts were derivatives of non-mDA progenitors in the donor cells including non-midbrain progenitors (P_vMeten_PDE1A) and midbrain non-mDA progenitors (P_MesenBP_SP5 and P_MesenBP_CRH), while other the off-target cells (e.g., VLMCs and Glut_NKX6-1) in the grafts had the same ancestors as the mDA neurons (Figure 12H).

In summary, by constructing the single-cell transcriptional landscape of mDA neuron differentiation in vitro and graft composition in vivo, we revealed the developmental basis of both target and off-target neuronal cells that emerge during differentiation and after transplantation. Importantly, we identified specific surface markers representing authentic mDA progenitors, which could be used to generate highly purified target cells to achieve stable and predictable graft outcomes with improved therapeutic efficacy in the treatment of PD. The ability to produce homogeneous stem cell products represents a revolutionary step on the road toward safer, more effective stem cell therapies. Further efforts are also needed to evaluate the long-term therapeutic efficacy of marker-sorted progenitors by finer behavioral assessment in nonhuman primate PD models.

## Methods

### Experimental animals.

SCID beige mice (male and female) were purchased from Vital River Laboratories. All animals used in this study were group-housed in a 12-hour light/12-hour dark cycle with ad libitum access to food and water.

### CRISPR/Cas9-mediated genome editing and generation of hESC lines.

To establish reporter cell lines, guide RNAs targeting the first 100 bp of the 3′ homology arm were designed using web-based tools designed by the Zhang laboratory (https://zlab.bio/guide-design-resources and https://www.benchling.com/). Donor plasmids were designed with the following structure: (a) the 5′ homology arm included the last approximately 1000 bp of the last exon of the selected gene (before the stop codon); (b) P2A and sequences encoding a fluorescent protein were inserted in frame before the stop codon of the targeted gene; (c) human GH polyA, mouse PGK promoter, puromycin or neomycin resistance gene, and polyA sequences were inserted following P2A and tdT; and (d) the 3′ homology arm included a stop codon and the next approximately 1000 bp. Details of the design and construction of the knock-in cell lines are provided in Supplemental Methods.

### Generation of midbrain dopaminergic neurons.

hPSCs (1 day after passaging) on irradiated MEFs were cultured in neural induction medium consisting of DMEM/F12, 1% N2 supplement (Gibco, Thermo Fisher Scientific), 1× MEM Non-Essential Amino Acids Solution in the presence of 2 μM SB431542 (Stemgent), 2 μM DMH-1 (Tocris), 500 ng/mL sonic hedgehog (SHH) (C25II, R&D Systems), and 0.4 μM CHIR99021 (Tocris) for 8 days. On day 9, individual colonies were gently blown off after they were rinsed with dispase and passaged onto irradiated MEFs. From days 9 to 12, the colonies were cultured in neural induction medium containing 100 ng/mL SHH (C25II), 0.4 μM CHIR99021, and 1 μM smoothened agonist (SAG) (MilliporeSigma). On day 13, individual colonies were gently blown off and expanded as floating clusters in suspension in the neural induction medium containing 0.5 μM SAG, 20 ng/mL SHH (C25II), and 100 ng/mL FGF8b (PeproTech) for 9 days (days 13–21). From days 22 to 36, neurospheres were cultured in neural induction medium containing 20 ng/mL SHH (C25II) and 20 ng/mL FGF8b. After day 36, neurospheres were committed to neuronal maturation in neurobasal medium supplemented with 1% N2, 2% B-27 Supplement (Life Technologies, Thermo Fisher Scientific), 10 ng/mL brain-derived neurotrophic factor (BDNF, PeproTech), 10 ng/mL glial-derived neurotrophic factor (GDNF) (PeproTech), 200 μM ascorbic acid (MilliporeSigma), 1 μM cAMP (MilliporeSigma), and 0.5 ng/mL transforming growth factor β3 (TGF-β3) (R&D Systems). 0.5 μM Rho-kinase (ROCK) inhibitor (Merck Millipore) and 10% B-27 Supplement without Vitamin A (Life Technologies, Thermo Fisher Scientific) were added to improve cell survival during passaging. For day-51 cultures used for scRNA-Seq, neurospheres were committed to terminal differentiation by additional supplementation with 10 μM DAPT (Tocris). Additionally, it is worth mentioning that we did not selectively blow off bulging clones on day 9 and did not pipette-blow neurospheres every time we changed the medium to eliminate dead cells or attached cells as was previously done (22). Instead, we blew off all visible clones on day 9 and kept the neurospheres intact to mimic an industrial manufacturing process.

### PD model and cell transplantation.

Surgical procedures for producing a model of PD in SCID mice were performed as described previously (8). SCID mice (7–8 weeks old) were anesthetized with 2% isoflurane mixed in oxygen. Then, 1 μL of 6-OHDA (3 mg/mL, in saline with 1% ascorbic acid, MilliporeSigma) was injected into the left substantial nigra (anterior-posterior [AP] = –2.9 mm, lateral [L] = –1.1 mm, vertical [V] = –4.5 mm, from skull). Animals were randomly grouped and transplanted with mDA progenitors or aCSF (control). Cells (100,000 or 7500) were resuspended in 1 μL aCSF containing 0.5 mM Rock inhibitor, 2% B-27 Supplement without Vitamin A, and 20 ng/mL brain-derived neurotrophic factor (BDNF), and then injected into the left striatum (A*P =* +0.6 mm, L = –1.8 mm, V = –3.2 mm, from dura).

### Amphetamine-induced rotation test and analysis.

Four weeks after lesioning, lesion severity was assessed using the amphetamine-induced rotation test (amphetamine dissolved in saline, 5 mg/kg, i.p.). Beginning 10 minutes after the amphetamine injection, motor behavior was recorded for 120 minutes using SmartPSS software. The videos were analyzed manually. Ipsilateral and contralateral rotations were counted. Data are presented as the net ipsilateral rotation within 60 minutes. Animals displaying a behavioral deficit (>300 rotations in 60 minutes) were defined as successful PD models and used for cell transplantation. The behavioral test was conducted 2, 4, and 6 months after transplantation.

### scRNA-Seq using the 10x Genomics chromium platform.

For in vitro differentiated samples, attached colonies (day 8, stage I) or neurospheres (day 14, stage II; day 21, stage III; day 28, stage IV; day 35, stage V) were digested using TrypLE Express Enzyme for 10 minutes at 37°C and washed twice with neural induction medium. Cells were then passed through a 35 μm cell sieve (BD) to obtain a single-cell suspension. Chromium Single Cell 3′ Reagent Kits (version 2) were used for library preparation (10x Genomics). Libraries were sequenced on an Illumina Hiseq PE150. Details on scRNA-Seq of graft samples are provided in Supplemental Methods.

hESC maintenance, feeder-free hESC maintenance, calcium phosphate transfection, cell sorting and flow cytometric analysis, tissue preparation and immunohistochemical analysis, imaging and quantification, whole-cell patch-clamp recording of brain slices, and processing and analysis of scRNA-Seq and bulk RNA-Seq data sets are described in the Supplemental Methods.

### Data and code availability.

The RNA-Seq and processed data reported in this work have been deposited in the NCBI’s Gene Expression Omnibus (GEO) database (GEO GSE204795 and GSE204796). Scripts reproducing the analysis are available on GitHub (https://github.com/MichaelPeibo/mDA-time-course-scRNA-seq; branch name: paper-analysis, commit ID: ab3df1839833ea235497ec1508533c34ddfffa52).

### Statistics.

Data are presented as the mean ± SEM. In all studies, statistical analyses included multiple unpaired, 2-tailed *t* tests with Holm-Šidák correction, 1-way ANOVA with Tukey’s multiple-comparison test, and 2-way ANOVA followed by Dunnett’s multiple-comparison test. A *P* value of less than 0.05 was considered statistically significant in all experiments.

### Study approval.

All animal experiments were conducted according to a protocol approved by the IACUC at the Institute of Neuroscience, CAS Center for Excellence in Brain Science and Intelligence Technology of the Chinese Academy of Sciences (Shanghai, China).

## Author contributions

YC conceived and supervised the project. PX constructed plasmids and transgenic cell lines and performed neurosphere immunostaining, scRNA-Seq experiments, and bioinformatics analyses. HH performed behavioral tests, histology experiments, and statistical analysis. Q Gao, PX, and MX performed cell differentiation analyses. Q Gao and HH performed cell transplantations. YZ performed electrophysiological recordings. YZ, ZW, and PX dissected mouse brains for graft scRNA-Seq. Xiao Zhang generated hiPSC lines. LS, Xiao Zhang, GH, ZW, ZY, Xinyue Zhang, and WZ helped construct plasmids and cell lines. Q Guan helped with animal experiments. PX, HH, and YC collected and analyzed data. PX and YC wrote the manuscript, and PX, YC, and MX edited the manuscript.

## Supplementary Material

Supplemental data

## Version 1 06/14/2022

In-Press Preview

## Version 2 07/15/2022

Electronic publication
