# Supplementary material for: Human midbrain dopaminergic neuronal differentiation markers predict cell therapy outcomes in a Parkinson’s disease model
Source: J Clin Invest. 2022 Jul 15;132(14):e156768. doi: 10.1172/JCI156768 (PMC9282930; doi:10.1172/JCI156768)
Supplement: Supplemental data [file jci-132-156768-s128.pdf]

SUPPLEMENTAL MATERIALS

Supplemental Figures 1-8

Supplemental Methods

Human midbrain dopaminergic neuronal differentiation markers predict cell therapy outcome in a Parkinson's disease model

Peibo Xu, Hui He, Qinqin Gao, Yingying Zhou, Ziyan Wu, Xiao Zhang, Linyu Sun, Gang Hu, Qian Guan, Zhiwen You, Xinyue Zhang, Wenping Zheng, Man Xiong, Yuejun Chen

Figure S1

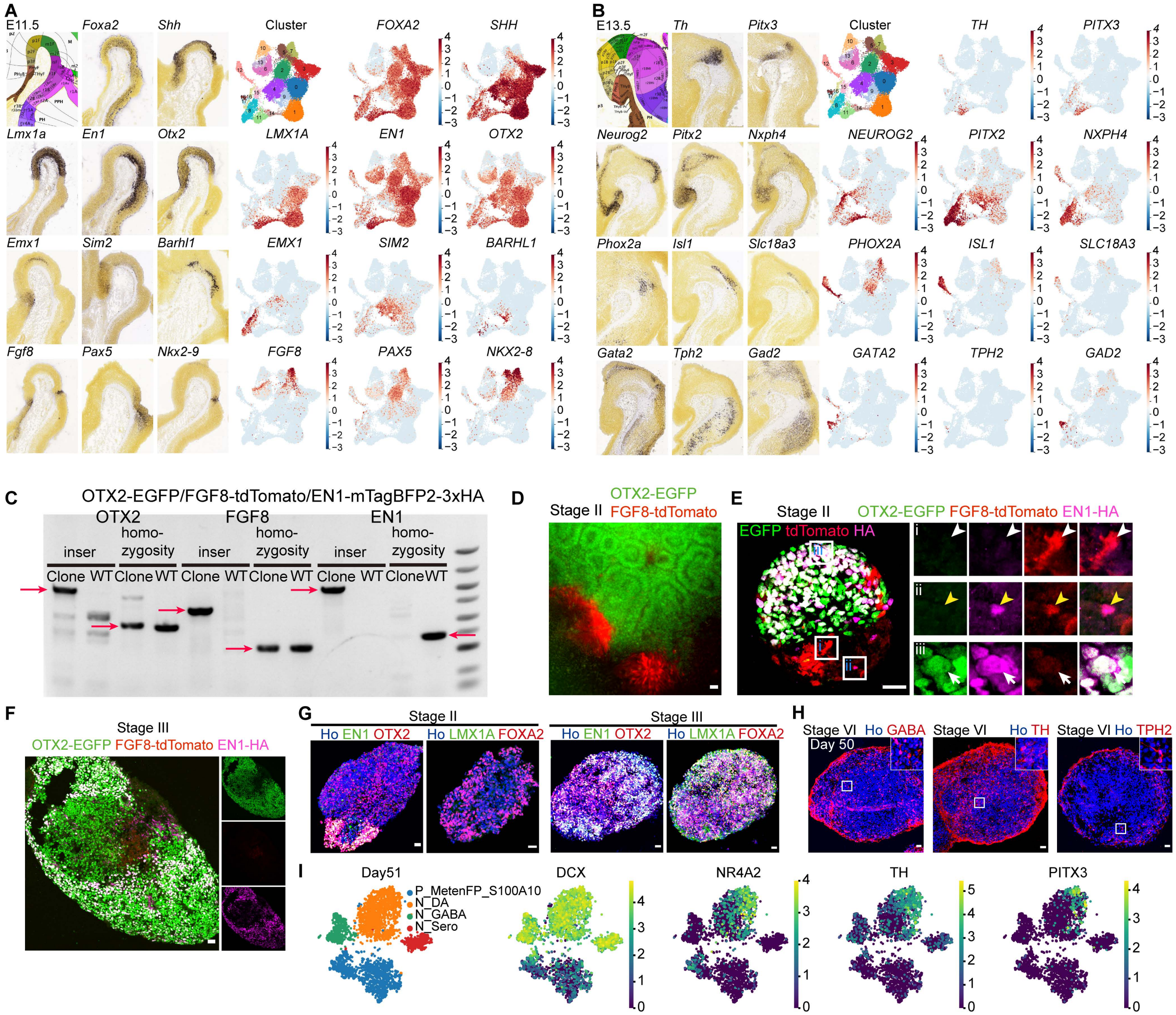

A
E11.5
Foxa2
Shh
Cluster
FOXA2
SHH
Lmx1a
En1
Otx2
LMX1A
EN1
OTX2
Emx1
Sim2
Barhl1
EMX1
SIM2
BARHL1
Fgf8
Pax5
Nkx2-9
FGF8
PAX5
NKX2-8
B
E13.5
Th
Pitx3
Cluster
TH
PITX3
Neurog2
Pitx2
Nxph4
NEUROG2
PITX2
NXPH4
Phox2a
Isl1
Slc18a3
PHOX2A
ISL1
SLC18A3
Gata2
Tph2
Gad2
GATA2
TPH2
GAD2
C
OTX2-EGFP/FGF8-tdTomato/EN1-mTagBFP2-3xHA
OTX2
FGF8
EN1
inser
homo-zygosity
Clone WT
D
Stage II
OTX2-EGFP
FGF8-tdTomato
E
Stage II
OTX2-EGFP FGF8-tdTomato EN1-HA
EGFP tdTomato HA
i
ii
iii
F
Stage III
OTX2-EGFP FGF8-tdTomato EN1-HA
G
Stage II
Stage III
Ho EN1 OTX2
Ho LMX1A FOXA2
H
Stage VI Ho GABA
Day 50
Stage VI Ho TH
Stage VI Ho TPH2
I
Day51
P_MetenFP_S100A10
N_DA
N_GABA
N_Sero
DCX
NR4A2
TH
PITX3

Supplemental Figure 1. Immunocytochemistry of cell types in neurospheres and reporter cell line, comparison with developing midbrain.

(A and B) In situ hybridization (left) and gene expression (right) of selected genes at E11.5 (A) and E13.5 (B). Note that Nkx2-9 in mouse is known as NKX2-8 in human. (C) PCR genotyping of the OTX2-EGFP/FGF8-tdT/EN1-mTagBFP2-3xHA hPSC line. Red arrows indicate expected PCR products. For OTX2-EGFP left-arm (LA) and homozygous, FGF8-tdT LA and homozygous, EN1-mTagBFP-3xHA LA and homozygous, the expected PCR product is ~2000 bp and ~900 bp, ~1300 bp and ~1000 bp, ~2000 bp and ~700 bp, respectively. Clones with expected insertion PCR products have correct insertion of donor plasmid. Clones without expected homozygosity PCR products are homozygous and with expected PCR products are heterozygous. H9 ESCs (WT) is included as a control. (D) Typical cell colony of stage II EGFP/tdT by live imaging. Scale bar, 50 μm. (E) Neurospheres of stage II by immunostaining EGFP/tdT/HA-tag. The white arrowhead indicates an FGF8-tdT$^+$ cell. The white arrow indicates an OTX2-EGFP$^+$/EN1-HA$^+$ cell. The yellow arrowhead indicates a FGF8-tdT$^+$/EN1-HA$^+$ cell. Scale bar, 25 μm. (F) Neurospheres of stage III by immunostaining EGFP/tdT/HA-tag. Scale bar, 25 μm. (G and H) Neurospheres were immunostained with mDA progenitor markers at stage II and III (G) (EN1, OTX2, LMX1A, and FOXA2) or neuronal markers (H) (GABA, TH, and TPH2) at stage VI (day 50). Scale bar, 25 μm. (I) scRNA-seq of mDA neuron differentiation in vitro at day 51 and gene expression of pan-neuronal marker *DCX*, mDA marker *NR4A2*, *TH* and *PITX3* on umap embeddings.

Figure S2

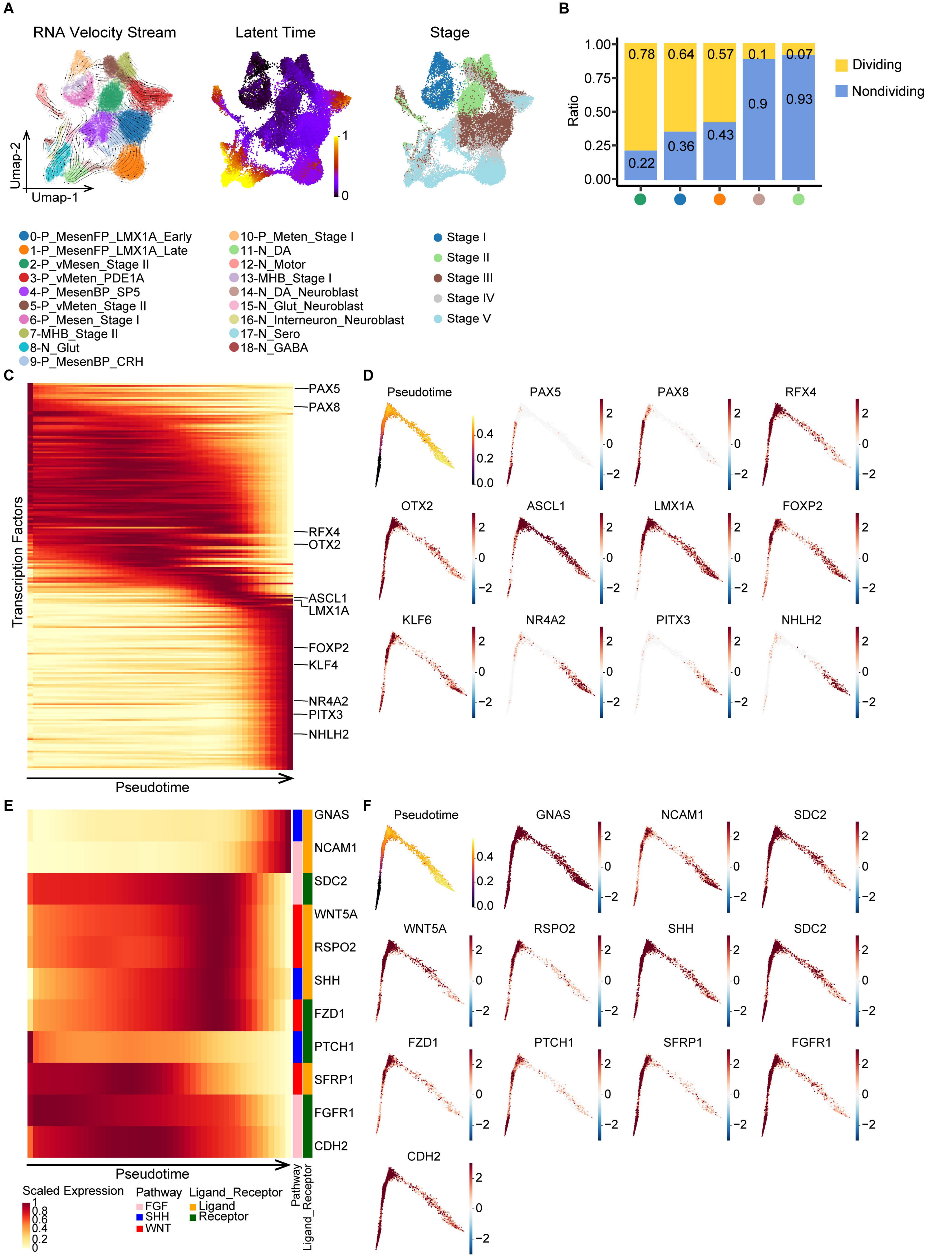
A
RNA Velocity Stream
Latent Time
Stage
Umap-2
Umap-1
1
0
0-P_MesenFP_LMX1A_Early
1-P_MesenFP_LMX1A_Late
2-P_vMesen_Stage II
3-P_vMeten_PDE1A
4-P_MesenBP_SP5
5-P_vMeten_Stage II
6-P_Mesen_Stage I
7-MHB_Stage II
8-N_Glut
9-P_MesenBP_CRH
10-P_Meten_Stage I
11-N_DA
12-N_Motor
13-MHB_Stage I
14-N_DA_Neuroblast
15-N_Glut_Neuroblast
16-N_Interneuron_Neuroblast
17-N_Sero
18-N_GABA
Stage I
Stage II
Stage III
Stage IV
Stage V
B
Ratio
1.00
0.75
0.50
0.25
0.00
0.78
0.64
0.57
0.1
0.07
0.22
0.36
0.43
0.9
0.93
Dividing
Nondividing
C
Transcription Factors
PAX5
PAX8
RFX4
OTX2
ASCL1
LMX1A
FOXP2
KLF4
NR4A2
PITX3
NHLH2
Pseudotime
D
Pseudotime
PAX5
PAX8
RFX4
OTX2
ASCL1
LMX1A
FOXP2
KLF6
NR4A2
PITX3
NHLH2
E
GNAS
NCAM1
SDC2
WNT5A
RSPO2
SHH
FZD1
PTCH1
SFRP1
FGFR1
CDH2
Pseudotime
Pathway
Ligand_Receptor
Scaled Expression
FGF
SHH
WNT
Ligand
Receptor
F
Pseudotime
GNAS
NCAM1
SDC2
WNT5A
RSPO2
SHH
SDC2
FZD1
PTCH1
SFRP1
FGFR1
CDH2

Supplemental Figure 2. Gene cascade of transcription factors, ligands and receptors of selected pathways.

(A) Visualization using umap embeddings by all clusters RNA velocity stream, latent time, and stages. (B) Bar plot shows the ratio of dividing cells and nondividing cells of each mDA-related cluster. (C and D) Transcription factors (TFs) expression cascade during differentiation of mDA neuron. (C) Heatmap showing transcription factors expression along pseudotime, which are selected from genes in Figure 4C. Typical TFs are listed at the right of the heatmap. (D) Typical TFs expression on diffusion map. (E) Varying ligands and receptors gene expression cascade during differentiation of mDA neuron. Three pathways (SHH, FGF, and WNT) were used to select varying ligands and receptors. (F) Gene expression of ligands and receptors from (E) on diffusion map.

Figure S3

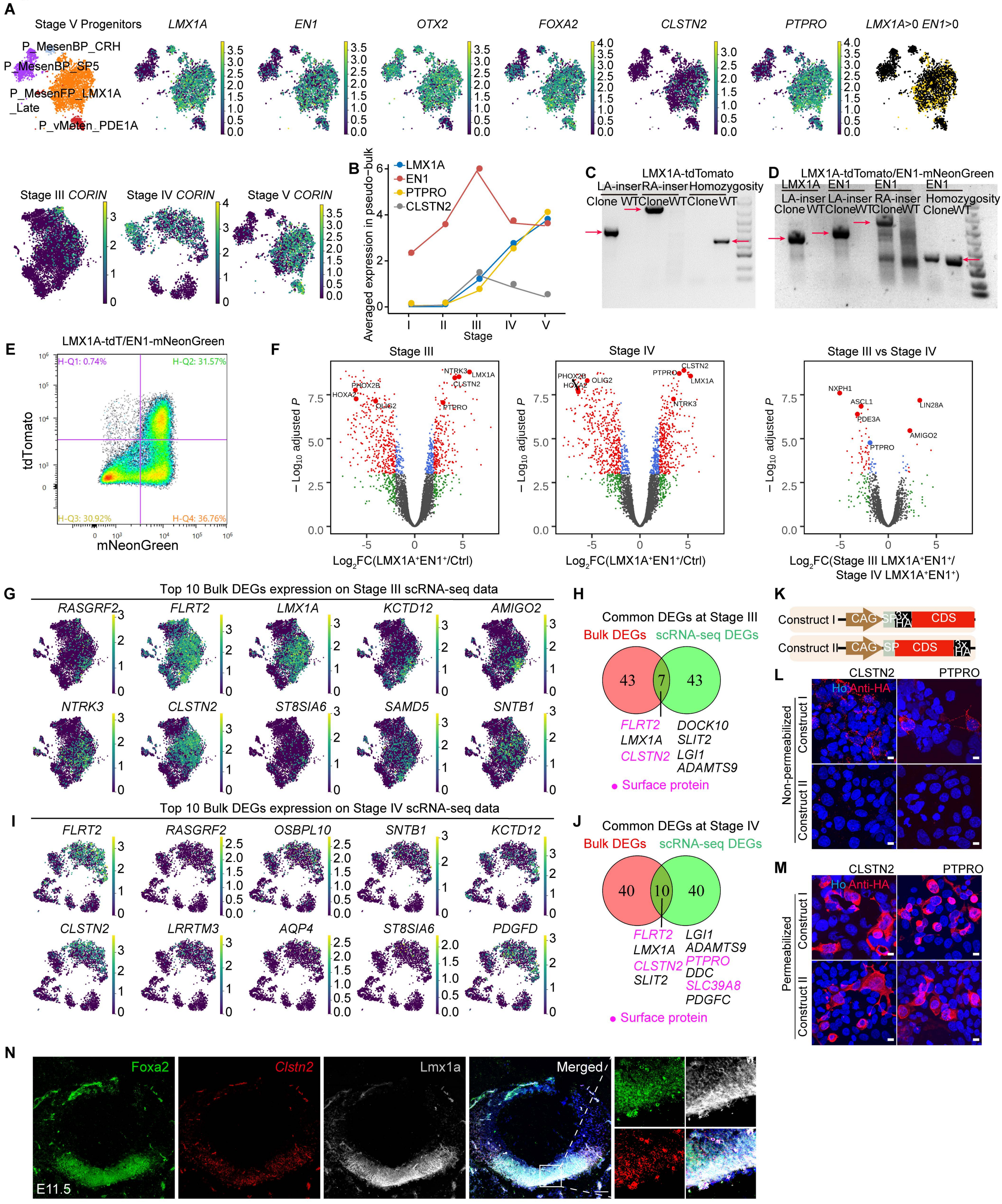

A
Stage V Progenitors
LMX1A
EN1
OTX2
FOXA2
CLSTN2
PTPRO
LMX1A>0 EN1>0
P_MesenBP_CRH
P_MesenBP_SP5
P_MesenFP_LMX1A_Late
P_vMeten_PDE1A
Stage III CORIN
Stage IV CORIN
Stage V CORIN
B
Averaged expression in pseudo-bulk
Stage
LMX1A
EN1
PTPRO
CLSTN2
C
LMX1A-tdTomato
LA-inser
RA-inser
Homozygosity
Clone WT
D
LMX1A-tdTomato/EN1-mNeonGreen
E
LMX1A-tdT/EN1-mNeonGreen
H-Q1: 0.74%
H-Q2: 31.57%
H-Q3: 30.92%
H-Q4: 36.76%
tdTomato
mNeonGreen
F
Stage III
Stage IV
Stage III vs Stage IV
− Log10 adjusted P
Log2FC(LMX1A+EN1+/Ctrl)
Log2FC(Stage III LMX1A+EN1+/Stage IV LMX1A+EN1+)
NTRK3
LMX1A
CLSTN2
PTPRO
PHOX2B
HOXA2
OLIG2
NXPH1
ASCL1
PDE3A
LIN28A
AMIGO2
G
Top 10 Bulk DEGs expression on Stage III scRNA-seq data
RASGRF2
FLRT2
LMX1A
KCTD12
AMIGO2
NTRK3
CLSTN2
ST8SIA6
SAMD5
SNTB1
H
Common DEGs at Stage III
Bulk DEGs
scRNA-seq DEGs
43
7
43
FLRT2
LMX1A
CLSTN2
DOCK10
SLIT2
LGI1
ADAMTS9
• Surface protein
I
Top 10 Bulk DEGs expression on Stage IV scRNA-seq data
FLRT2
RASGRF2
OSBPL10
SNTB1
KCTD12
CLSTN2
LRRTM3
AQP4
ST8SIA6
PDGFD
J
Common DEGs at Stage IV
Bulk DEGs
scRNA-seq DEGs
40
10
40
FLRT2
LMX1A
CLSTN2
SLIT2
LGI1
ADAMTS9
PTPRO
DDC
SLC39A8
PDGFC
• Surface protein
K
Construct I
CAG
SP
3X HA
CDS
Construct II
L
CLSTN2
PTPRO
Ho Anti-HA
Non-permeabilized
M
Permeabilized
N
Foxa2
Clstn2
Lmx1a
Merged
E11.5

Supplemental Figure 3. Construction of dual-reporter cell line and examining bulk RNA-seq DEGs on scRNA-seq data.

(A) Stage V gene expression (top), similar as Figure 5A. *CORIN* expression at stage III, IV and V progenitor clusters (bottom). (B) Selected marker averaged expression in pseudo-bulk by each stage. (C and D) PCR genotyping of the LMX1A-tdT (C) and LMX1A-tdT/EN1-mNeonGreen hPSC lines (D). *LMX1A* locus insertion PCR product for left-arm (LA) and right-arm (RA) are ~1500 bp and ~3000 bp, respectively. Homozygous, ~1000 bp. *EN1* locus insertion PCR product for LA and RA are ~1700 bp and ~3300 bp, respectively. Homozygous, ~700 bp. (E) Typical FACS image at stage III of mDA neuron differentiation using LMX1A-tdT/EN1-mNeonGreen hPSC line. (F) Volcano plots showing DEGs (stage III LMX1A$^+$EN1$^+$ versus control cells, stage IV LMX1A$^+$EN1$^+$ versus control cells, and stage III LMX1A$^+$EN1$^+$ versus stage IV LMX1A$^+$EN1$^+$ cells). Assigned red dots DEGs were determined using threshold: Log2 fold change = 2, p value cutoff = 10^{-3}. Enlarged red dots are representative cell type markers (*LMX1A*, *HOXA2*, *PHOX2B*, and *OLIG2*) or discovered surface markers (*CLSTN2*, *PTPRO*, and *NTRK3*). The enlarged blue dot *PTPRO* is significant based on p-value, while not reaching log2 fold change threshold. (G-J) The top 10 bulk DEGs (stage III or IV LMX1A$^+$EN1$^+$ versus other cells) projected onto stage III (G) and stage IV scRNA-seq data (I). (H, J) Venn diagrams show overlapped DEGs between the top 50 DEGs of double-positive bulk RNA-seq and scRNA-seq mDA progenitor cluster. (K) Diagram of two plasmids constructs. SP represents signal peptides of selected surface markers. (L and M) Immunocytochemistry of 293T cells transfected of construct I (top) and construct II (down). Anti-HA live staining (L) and fixed-permeabilized staining (M). Scale bar, 25 µm. (N) RNA FISH of *Clstn2* following immunohistochemistry by co-labeling Foxa2 and Lmx1a in E11.5 mouse mesencephalon. The zoomed views indicate magnified images of Foxa2$^+$Lmx1a$^+$*Clstn2*$^+$ progenitors. Scale bars, 100 µm.

Figure S4

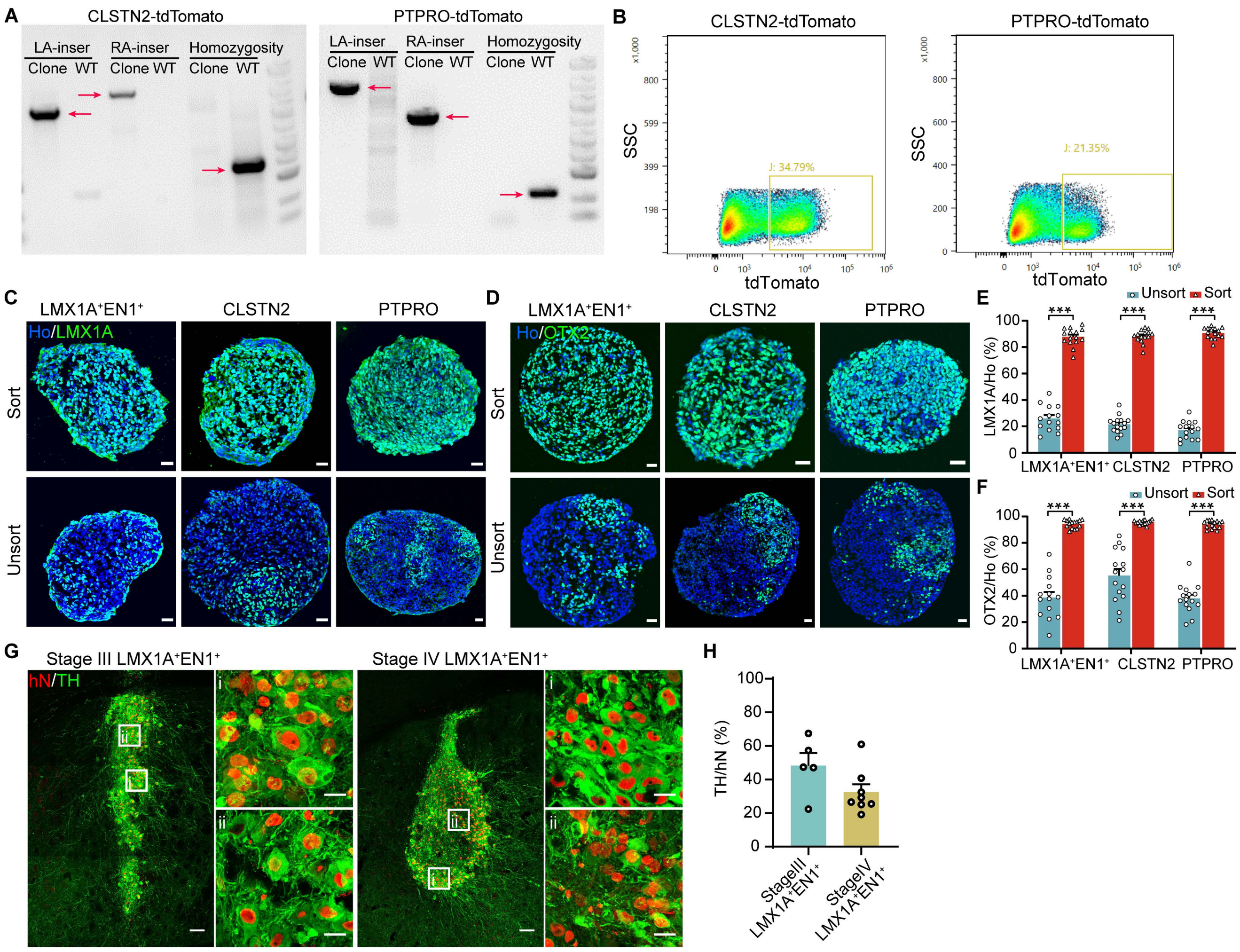
A
CLSTN2-tdTomato
LA-inser
RA-inser
Homozygosity
Clone WT
PTPRO-tdTomato
B
CLSTN2-tdTomato
PTPRO-tdTomato
SSC
tdTomato
J: 34.79%
J: 21.35%
C
LMX1A+EN1+
CLSTN2
PTPRO
Ho/LMX1A
Sort
Unsort
D
Ho/OTX2
E
Unsort
Sort
LMX1A/Ho (%)
F
OTX2/Ho (%)
G
Stage III LMX1A+EN1+
Stage IV LMX1A+EN1+
hN/TH
H
TH/hN (%)
StageIII LMX1A+EN1+
StageIV LMX1A+EN1+

Supplemental Figure 4. Enriching mDA progenitors in vitro.

(A) PCR genotyping of the CLSTN2- and PTPRO-tdT hPSC lines. The expected *CLSTN2* locus insertion PCR products for LA and RA are ~2200 bp and ~2800 bp, respectively. Homozygous, ~680 bp. *PTPRO* locus, LA, ~3000 bp; RA, ~1600 bp; homozygous, ~300 bp. (B) Typical FACS images at stage III (CLSTN2) and stage IV (PTPRO) of mDA neuron differentiation using CSLTN2- or PTPRO-tdT hPSC line. (C-F) Neurospheres in vitro immunostained with progenitor markers LMX1A (C) and OTX2 (D). Quantification of LMX1A^{+} (E) and OTX2^{+} cells (F) ratio in neurospheres. Scale bar, 25 µm. Multiple unpaired t test with Holm-Sidak correction. (G) Stage III LMX1A^{+}EN1^{+} progenitor- and stage IV LMX1A^{+}EN1^{+} progenitor-derived graft, immunostained with human nuclei (hN) and TH. Scale bar, 100 µm. (i) and (ii) represent the edge and center area of graft, respectively. Scale bar, 20 µm. (H) Quantification of TH^{+} neurons ratio in graft. N = 5 (stage III LMX1A^{+}EN1^{+}) and 8 (stage IV LMX1A^{+}EN1^{+}).

Figure S5

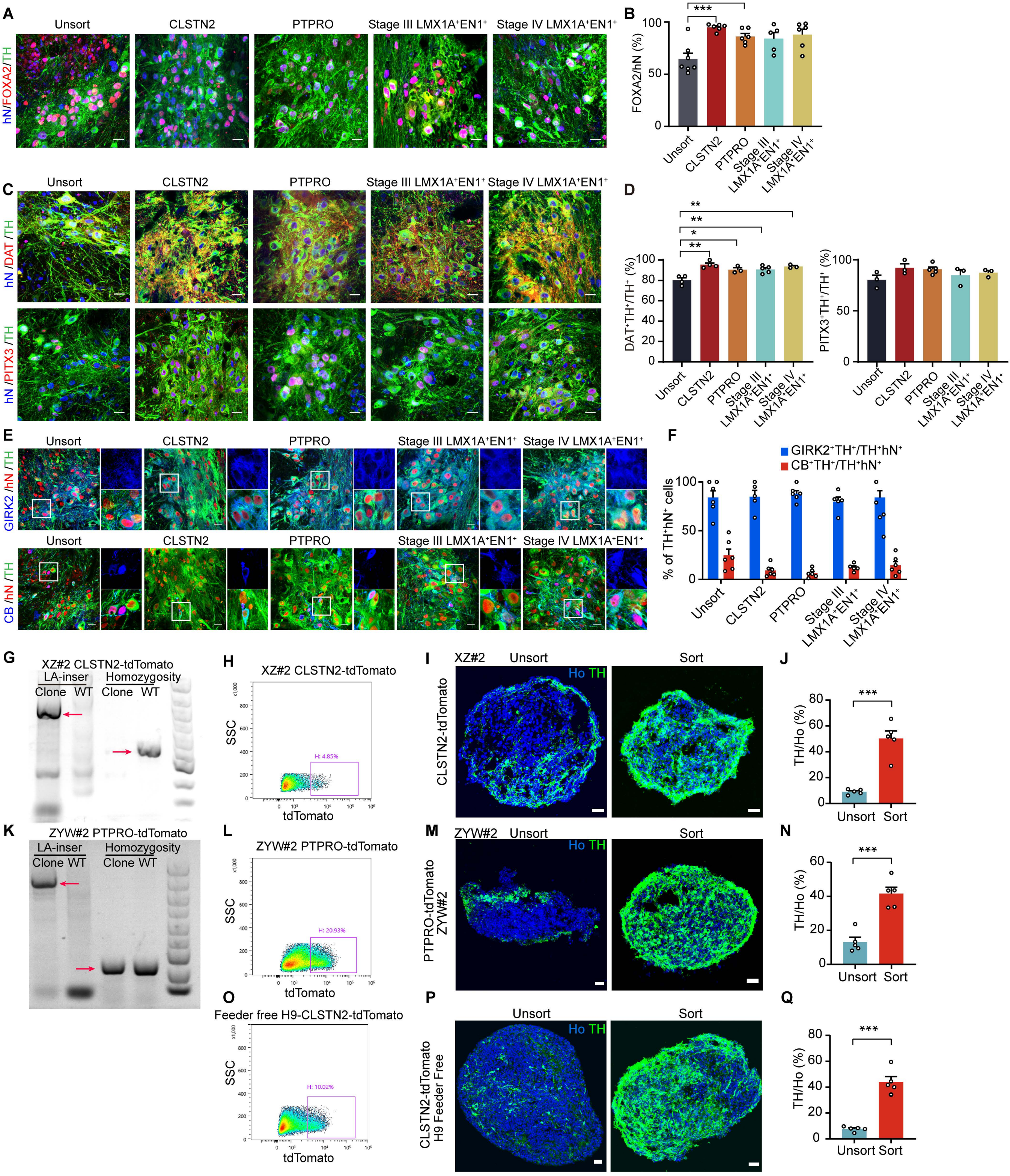

A
Unsort
CLSTN2
PTPRO
Stage III LMX1A+EN1+
Stage IV LMX1A+EN1+
hN/FOXA2/TH
B
FOXA2/hN (%)
C
hN /DAT /TH
hN /PITX3 /TH
D
DAT+TH+/TH+ (%)
PITX3+TH+/TH+ (%)
E
GIRK2 /hN /TH
CB /hN /TH
F
GIRK2+TH+/TH+hN+
CB+TH+/TH+hN+
% of TH+hN+ cells
G
XZ#2 CLSTN2-tdTomato
LA-inser
Homozygosity
Clone WT
H
XZ#2 CLSTN2-tdTomato
SSC
tdTomato
H: 4.85%
I
XZ#2
Sort
Ho TH
CLSTN2-tdTomato
J
TH/Ho (%)
K
ZYW#2 PTPRO-tdTomato
L
ZYW#2 PTPRO-tdTomato
H: 20.93%
M
ZYW#2
PTPRO-tdTomato ZYW#2
N
O
Feeder free H9-CLSTN2-tdTomato
H: 10.02%
P
CLSTN2-tdTomato H9 Feeder Free
Q

Supplemental Figure 5. Cellular identity of enriched mDA neuron and validation of mDA neuron enrichment in independent hiPS cell lines or feeder-free condition.

(A and B) Typical graft immunostained with FOXA2, hN and TH (A) and quantification of $FOXA2^+$ cells (B) ratio. Scale bar, 20 µm. N = 7 (Unsort), 6 (CLSTN2), 6 (PTPRO), 5 (stage III $LMX1A^+EN1^+$) and 6 (stage IV $LMX1A^+EN1^+$). One-way ANOVA followed by Tukey's multiple comparisons test. *p < 0.05, **p < 0.01, ***p < 0.001. (C) Graft immunostained with hN and DAT (top) or PITX3 (bottom). Scale bar, 20 µm. (D) Quantification of DAT^+TH^+/TH^+ cells (left) and $PITX3^+TH^+/TH^+$ cells (right) ratio. (E and F) Graft immunostained with hN and GIRK2 (top) or CB (bottom) and the quantification (F) of mDA subtypes in total TH^+ neurons ratio. Scale bar, 20 µm. Data are represented as mean ± SEM. *p < 0.05, **p < 0.01, ***p < 0.001. (G-Q) Validation of CLSTN2 (G-J) and PTPRO (K-N) at two independent hiPSC lines, and CLSTN2 at feeder-free culture condition (O-Q). PCR genotyping of the CLSTN2- (G) and PTPRO-tdT (K) hiPSC lines. The strategy of identification is the same as Supplemental Figure 4A. The corresponding mother cell line (XZ#2 hiPSC line, or ZYW#2 mononuclear cells, labeled as WT) is included as a control. Typical images of FACS at stage III (CLSTN2) of mDA neuron differentiation using CLSTN2-tdT hiPSC (H) or feeder-free H9 cell lines (O), or at stage IV (PTPRO) using PTPRO-tdT (L) hiPSC line. Typical mature neurospheres in vitro immunostained with TH (I, M, and P) and statistical analysis of TH ratio in sorted group and unsorted group (J, N, and Q) (1 batch, 5 neurospheres for each batch). Scale bar, 25 µm. Unpaired t test. Data are represented as mean ± SEM. ***p < 0.001.

Figure S6

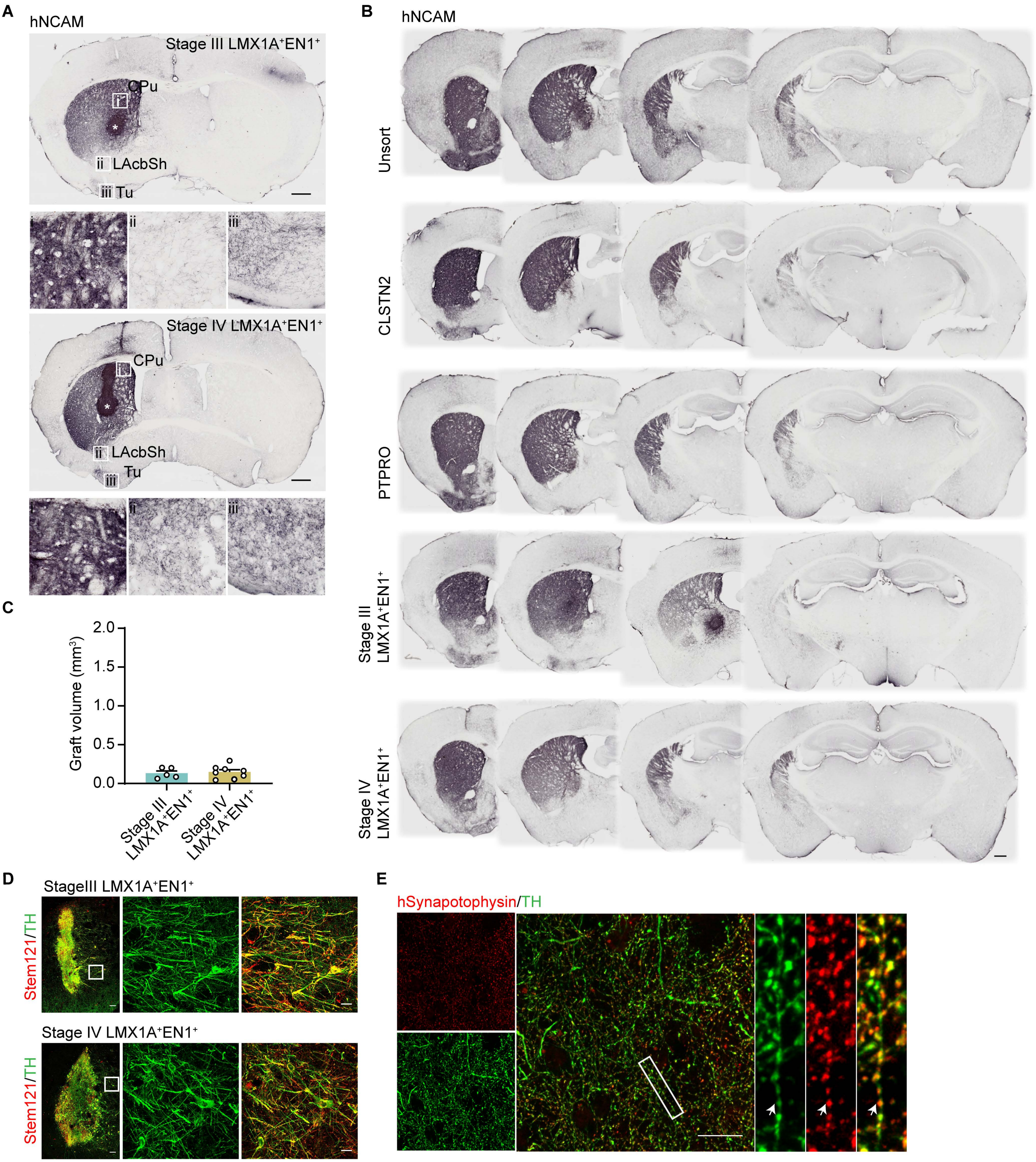
A
hNCAM
Stage III LMX1A+EN1+
CPu
i
ii LAcbSh
iii Tu
i
ii
iii
Stage IV LMX1A+EN1+
CPu
i
ii LAcbSh
Tu
iii
i
ii
iii
B
hNCAM
Unsort
CLSTN2
PTPRO
Stage III LMX1A+EN1+
Stage IV LMX1A+EN1+
C
Graft volume (mm3)
2.0
1.5
1.0
0.5
0.0
Stage III LMX1A+EN1+
Stage IV LMX1A+EN1+
D
StageIII LMX1A+EN1+
Stem121/TH
Stage IV LMX1A+EN1+
Stem121/TH
E
hSynapotophysin/TH

Supplemental Figure 6. Graft fiber innervation across different brain regions and synaptic integration of mDA neurons.

(A) $hNCAM^+$ fibers distribution and extension in stage III or IV $LMX1A^+EN1^+$ groups. The white asterisk indicates graft site. Scale bar, 500 μm. (B) Examination of hNCAM fiber extension across different brain regions. Scale bar, 500 μm. (C) The graft volumes at 6 months. N = 5 (stage III $LMX1A^+EN1^+$) and 8 (stage IV $LMX1A^+EN1^+$). (D) Grafts co-labeling human-specific fiber STEM121 and TH. Scale bar, 100 μm. Inset box represents zoomed view of extended graft fiber. Scale bar, 20 μm. (E) Typical immunohistochemistry images by co-labeling of human-specific synaptophysin and TH in CLSTN2-derived graft. Boxed areas are magnified on the right. White arrows indicate co-localization of human-specific synaptophysin with TH along the TH^+ fibers. Scale bar, 20 μm.

Figure S7

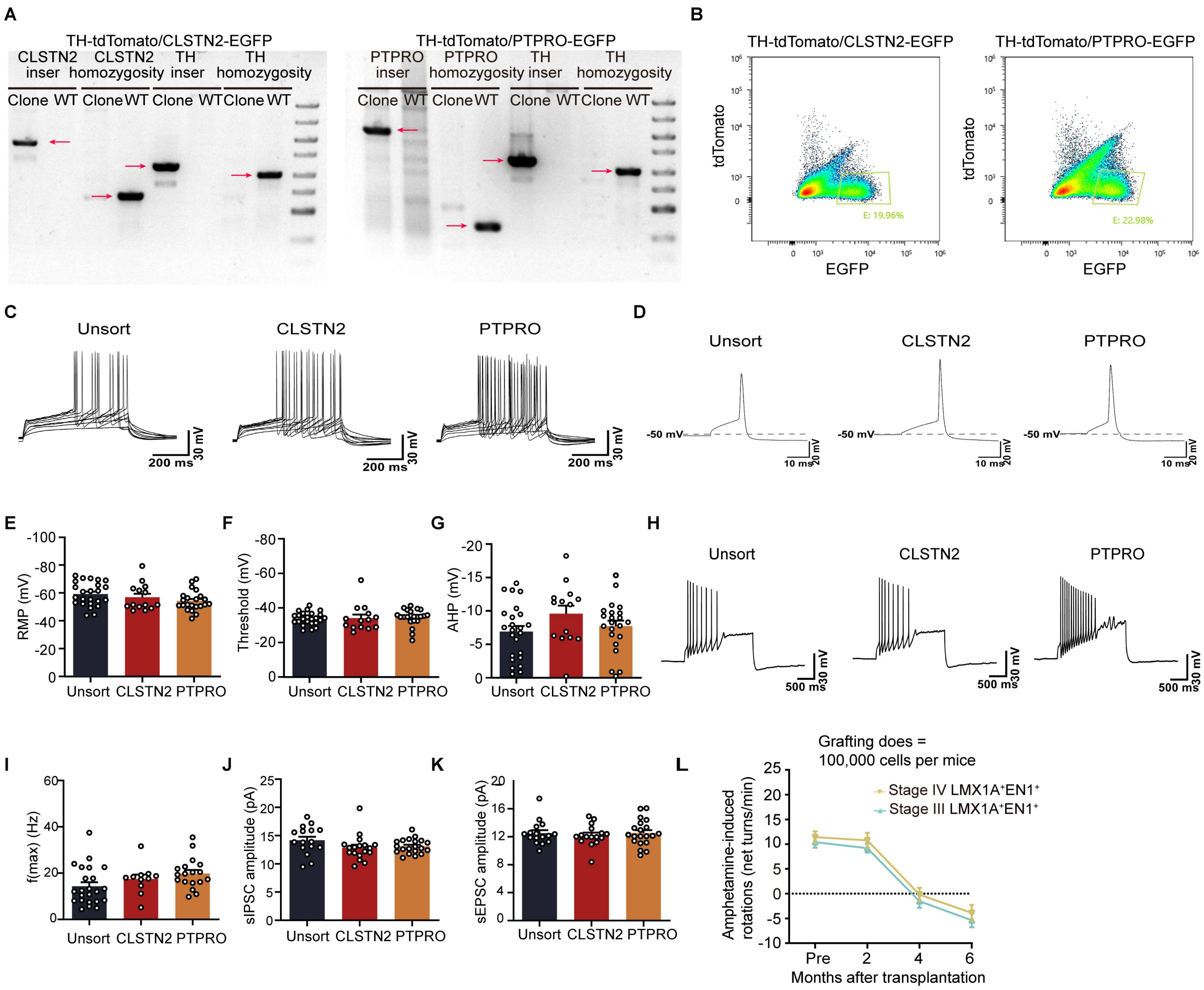

A
TH-tdTomato/CLSTN2-EGFP
CLSTN2 inser
CLSTN2 homozygosity
TH inser
TH homozygosity
Clone WT Clone WT Clone WT Clone WT
TH-tdTomato/PTPRO-EGFP
PTPRO inser
PTPRO homozygosity
TH inser
TH homozygosity
Clone WT Clone WT Clone WT Clone WT
B
TH-tdTomato/CLSTN2-EGFP
TH-tdTomato/PTPRO-EGFP
tdTomato
EGFP
E: 19.96%
E: 22.98%
C
Unsort
CLSTN2
PTPRO
200 ms
30 mV
D
Unsort
CLSTN2
PTPRO
-50 mV
10 ms
20 mV
E
RMP (mV)
Unsort CLSTN2 PTPRO
F
Threshold (mV)
Unsort CLSTN2 PTPRO
G
AHP (mV)
Unsort CLSTN2 PTPRO
H
Unsort
CLSTN2
PTPRO
500 ms
30 mV
I
f(max) (Hz)
Unsort CLSTN2 PTPRO
J
sIPSC amplitude (pA)
Unsort CLSTN2 PTPRO
K
sEPSC amplitude (pA)
Unsort CLSTN2 PTPRO
L
Grafting does = 100,000 cells per mice
Stage IV LMX1A⁺EN1⁺
Stage III LMX1A⁺EN1⁺
Amphetamine-induced rotations (net turns/min)
Pre 2 4 6
Months after transplantation

Supplemental Figure 7. TH-tdT/CLSTN2- and TH-tdT/PTPRO-EGFP cell lines validation and electrophysiological recording of grafted mDA neurons.

(A) PCR genotyping of the TH-tdT/CLSTN2-EGFP (left) and TH-tdT/PTPRO-EGFP (right) hPSC lines. Red arrows indicate expected PCR products. The expected PCR products for LA-based insertion and homozygosity identification correctly targeted TH locus are ~1200 bp and ~1000 bp, respectively. The expected PCR products for homozygosity identification correctly targeted *CLSTN2* locus and *PTPRO* locus are the same as Supplemental Figure 4A. The expected PCR products for LA-based insertion identification correctly targeted CLSTN2 and PTPRO locus are ~1700 bp and ~3000 bp, respectively. For homozygosity identification, clones without expected PCR products are homozygous and with expected PCR products are heterozygous. The mother cell line (H9 ESCs, labeled as WT) is included as a control. (B) Typical images of FACS at stage III (TH-tdT/CLSTN2-EGFP cell line, left) and stage IV (TH-tdT/PTPRO-EGFP cell line, right) of mDA neuron differentiation. (C and D) Typical traces of whole-cell patch-clamp recording of current-induced APs (C) and current-induced single AP (D) from grafted mDA neurons 5 months after transplantation. (E-G) Resting membrane potential (RMP) (E), threshold (F) and afterhyperpolarization (AHP) (G) from patched grafted mDA neurons. (H and I) Ramp current-induced APs (100–300 pA, duration 2000 ms) (H) and max frequency (I) of ramp current-induced APs from grafted mDA neurons. Recorded cell number, n = 23 (Unsort), 11 (CLSTN2), 18 (PTPRO). (J and K) The amplitude of sIPSCs (J) and sEPSCs (K). (L) Amphetamine-induced rotation behavior changes over 6 months post-transplantation. N = 9 (stage III LMX1A$^+$EN1$^+$) and 8 (stage IV LMX1A$^+$EN1$^+$).

Figure S8

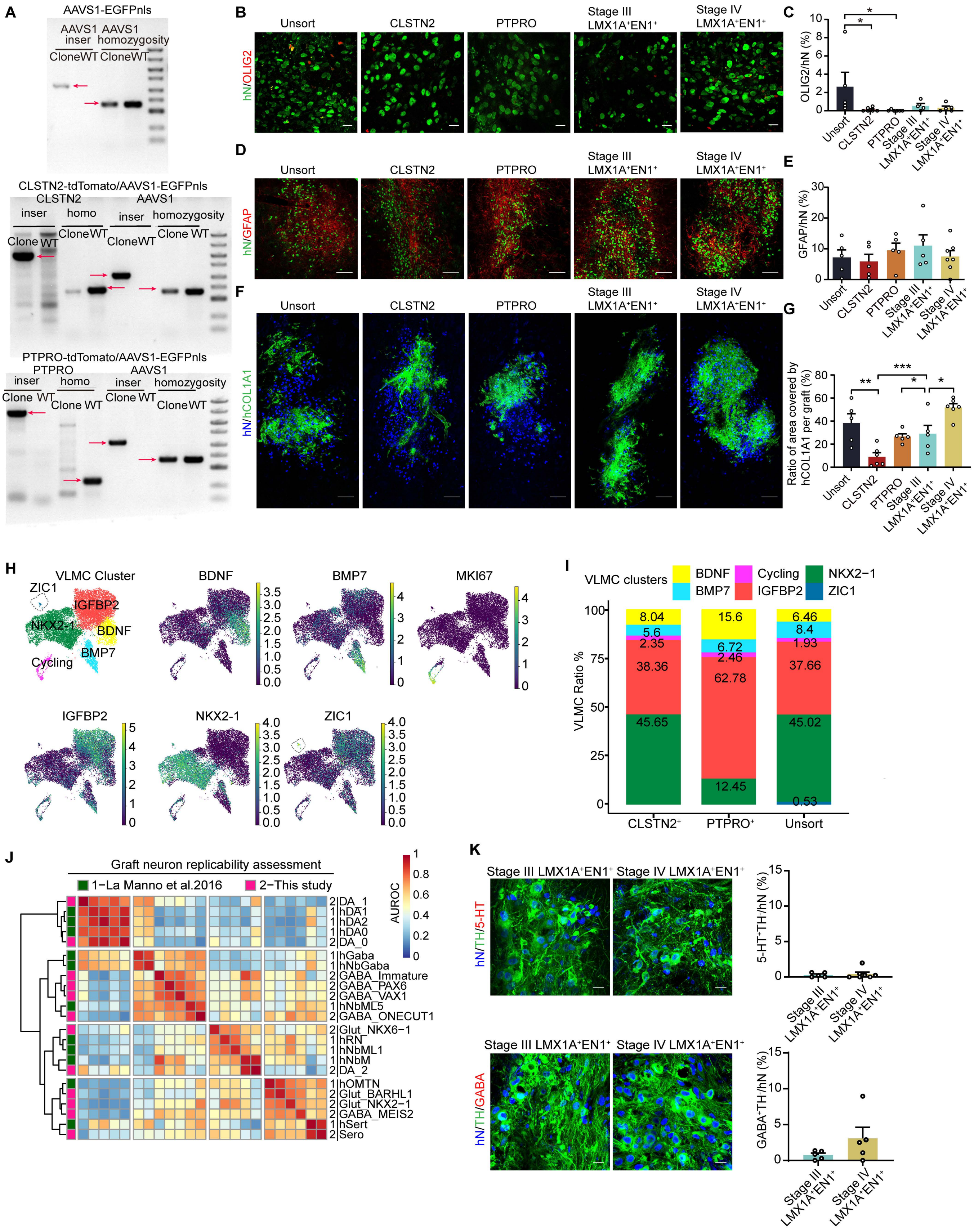

A
AAVS1-EGFPnls
AAVS1 inser
AAVS1 homozygosity
Clone WT
CLSTN2-tdTomato/AAVS1-EGFPnls
CLSTN2
AAVS1
inser
homo
homozygosity
PTPRO-tdTomato/AAVS1-EGFPnls
PTPRO
B
Unsort
CLSTN2
PTPRO
Stage III LMX1A⁺EN1⁺
Stage IV LMX1A⁺EN1⁺
hN/OLIG2
C
OLIG2/hN (%)
D
hN/GFAP
E
GFAP/hN (%)
F
hN/hCOL1A1
G
Ratio of area covered by hCOL1A1 per graft (%)
H
VLMC Cluster
ZIC1
IGFBP2
NKX2-1
BDNF
BMP7
Cycling
MKI67
I
VLMC clusters
VLMC Ratio %
CLSTN2⁺
PTPRO⁺
Unsort
J
Graft neuron replicability assessment
1−La Manno et al.2016
2−This study
AUROC
K
hN/TH/5-HT
5-HT⁺TH⁻/hN (%)
hN/TH/GABA
GABA⁺TH⁻/hN (%)

Supplemental Figure 8. Graft cell type composition.

(A) PCR genotyping of the AAVS1-EGFPnls, CLSTN2-, PTPRO-tdT/AAVS1-EGFPnls hPSC lines. PCR identification strategy for CLSTN2 and PTPRO locus is the same as Supplemental Figure 4A. The expected *AAVS1* locus PCR product for LA insertion is ~1500 bp; homozygous, ~600 bp. (B-G) Typical grafts were immunostained with oligodendrocyte or oligodendrocyte progenitor cell marker OLIG2 (B), astrocyte marker GFAP (D), VLMC marker COL1A1 (F), and hN in graft groups. Quantification of OLIG2^{+} cells (C) ratio, GFAP^{+} cells (E) ratio, and COL1A1^{+} density (G) per area in graft. Scale bar for (B), 20 μm. Scale bar for (D, F): 100 μm. One-way ANOVA followed by Tukey's multiple comparisons test. (H) Further clustering of VLMC group and representative marker expression of each VLMC subcluster. (I) VLMC subtype ratio by each graft group calculated using scRNA-seq data. (J) Heatmap of AUROC scores between graft neuronal clusters in this study and neuron-only data set from a public data set (4). (K) Typical images of graft, immunostained by 5-HT or GABA, and TH (left). Quantification of 5-HT^{+} and GABA $^{+}$ neurons ratio (right) in stage III or stage IV LMX1A^{+}EN1^{+} groups.

Supplemental Methods

Human embryonic stem cells. hPSCs (line H9, passages 20-50) were cultured on a six-well plate coated with irradiated mouse embryonic fibroblasts (MEFs). Cultures were maintained at 37°C in a humidified incubator with 5% CO2. Cells were maintained in Dulbecco's modified Eagle's medium/F12 (Gibco) that contained 20% Knockout serum replacement (Gibco), 1X MEM Non-Essential Amino Acids Solution (Life Technologies), 0.5X GlutaMAX (Gibco), and 0.1 mM 2-mercaptoethanol (Sigma). Recombinant human FGF-basic (10 ng/ml) was added when feeding cells. When cells expanded to 70% confluence (about 5 days), they were digested with Dispase (Gibco, 1 mg/ml) and then passaged to a new plate coated with irradiated MEFs.

Feeder-free hES cell maintenance. The CLSTN2-tdT hPSC line was switched into feeder-free conditions by seeding dissociated cells onto a six-well plate coated with vitronectin XF (Stemcell Technologies). Cells were fed daily with mTeSR1 medium (Stemcell Technologies) and passaged 5-6 days using TrypLE Express Enzyme (1X, Gibco). Cells were used for differentiation after 3-4 passages from transformation. The differentiation culture condition was the same as hPSCs on irradiated MEF feeder cells.

Construction of knock-in cell lines. For the OTX2-EGFP/FGF8-tdT/EN1-BFP-3x-HA-tag reporter line, the OTX2-EGFP hPSC line was first generated, then P2A and tdT

sequences were inserted in frame into an FGF8 donor plasmid with the same structure as OTX2-EGFP, but using homology arms of FGF8 and neomycin resistance gene. At the final step, P2A and BFP-3x-HA-tag were inserted in frame into the EN1 donor plasmid with the same structure of OTX2-EGFP but using homology arms of EN1 and puromycin resistance gene. For the LMX1A-tdT/EN1-mNeonGreen reporter line, the LMX1A-tdT hPSC line was first generated. P2A and mNeonGreen were then inserted in frame into the EN1 donor plasmid with the same structure of LMX1A-tdT donor plasmid but using neomycin resistance gene. For surface marker reporter cell lines, P2A and tdT were inserted in frame into the *CLSTN2* or *PTPRO* locus, with same structure of LMX1A-tdT donor plasmid using homology arms of CLSTN2 or PTPRO. To establish surface marker reporter/EGFPnls cell lines for scRNA-seq analysis of grafts, AAVS1-NeoR-CAG-EGFPnls-WPRE-polyA donor plasmids were constructed and electroporated into H9 ESCs or established surface marker reporter cell lines using transcription-activator-like effector nucleases (TALENs). We constructed TH-tdT/Surface marker-EGFP reporter cell lines based on TH-tdT that our lab previously generated (1). Briefly, we modified the surface marker-tdT donor plasmid by replacing tdT with EGFP but used the neomycin resistance gene. Targeting guide RNAs were the same as the surface marker-tdT. Electroporation, genomic DNA extraction, and genomic PCR identification were performed as described (1, 2). Single guide RNA sequences for each donor plasmid are listed below. SgRNA human TH-1: GAGGTTGGGAAGGGCCCTCA, sgRNA human TH-2: CCTGCACTGTCCCGGAGCTC; sgRNA human LMX1A-1:

CATATTCTTTGAGGGGTCAC; sgRNA human LMX1A-2: GCCTAGTCACAGAACTCTAG; sgRNA human EN1-1: ACGACGGCGGCGGTGCCGGG; sgRNA human EN1-2: GCACCGCCGCCGTCGTCTCC; sgRNA human OTX2-1: TAACTCTTTTAACCAATGCC; sgRNA human OTX2-2: CATCTGATCAAAGTTCCGAG; sgRNA human FGF8-1: CTGGCATTGTGGGGAGGGCC; sgRNA human FGF8-2: CGCAGAGAGGCTCATCCTGT; sgRNA human CLSTN2-1: AGGTGAGGTATGATAGACAT; sgRNA human CLSTN2-2: TGTCTGTGACATGTCTGGGA; sgRNA human PTPRO-1: TTCTAAGACTGAGCACTCG; sgRNA human PTPRO-2: ACTATTAGAGGGGGATGTGA.

Calcium phosphate transfection of surface protein plasmid construct. 293T cells were seeded onto 24-well plates at a density of 40,000 cells per well. Transfection was performed 20 hours after cell seeding. The transfection mixture per well included 500 ng of plasmids constructs (construct I: pCAG-SP-3x-HA-surface protein CDS; construct II: pCAG-SP-surface protein CDS-3x-HA), 5 µl 2M $CaCl_2$, and sterile H_2O added to 18.5 µl. Then, 18.5 µl 2X Hepes Buffered Saline (HBS) was added dropwise to the transfection mixture while bubbling air with another pipette. The medium was changed 8 hours after transfection.

Cell sorting and flow cytometric analysis. Neurospheres were dissociated into single cells with StemPro Accutase (Gibco) for 8 minutes at room temperature (RT). The sorted cells were seeded onto Lipidure-CM5206 (NOF CORPORATION) coated 96 Well Conical (V) Bottom Plate (Thermo Scientific) at a density of 10, 000 cells/well. The analysis was performed on a BD LSRFortessa flow cytometer (BD, USA) or MA900 Multi-Application Cell Sorter (Sony, Japan), and data were further analyzed using Cell Sorter software. 0.5 μM ROCK inhibitor (Sigma) and 10% B-27 Supplement without vitamin A were added to improve cell survival when reseeded.

For stage IV-sorted cell lines, neurospheres were matured into neurons in neurobasal medium supplemented with 1% N2, 2% B-27 Supplement, 10 ng/ml BDNF, 10 ng/ml GDNF, 200 μM ascorbic acid, 1 μM cAMP, and 0.5 ng/ml TGF-β3. For stage III-sorted cell lines, neurospheres were cultured in neural induction medium containing 20 ng/ml SHH (C25II), 20 ng/ml FGF8b, and Penicillin-Streptomycin (5,000 U/mL, Gibco) until day 30. They were then matured into neurons. For correlation analysis of progenitor marker ratio and neuron ratio, CLSTN2- or PTPRO-EGFP/TH-tdT cell lines were used to perform VM differentiation. The ratio was estimated by flow cytometric analysis on stage III (CLSTN2-EGFP) or stage IV (PTPRO-EGFP) for progenitor ratio, and at day 50 (TH-tdT) for neuron ratio.

Tissue preparation and immunohistochemistry. Cultured neurospheres were collected on day 30 (for progenitor) or day 45 (for neuron) and fixed in 4%

paraformaldehyde (PFA) dissolved in culture medium for 15-20 minutes at RT based on the size of the organoids. Neurospheres were washed with DPBS every 10 minutes, three times in total, and then incubated in 30% sucrose in PB at 4°C until the organoids sank to the bottom of the centrifuge tube. Neurospheres were subsequently embedded in Optimal Cutting Temperature (OCT) embedding media and 14-μm sections were cut using a cryostat (Thermo Scientific™ HM525 NX). Sections were collected on Premiere 9308W slides for labeling. Labeling procedures are described below.

For mouse brains, once behavioral data had been collected, animals were deeply anesthetized and transcardially perfused with 0.9% saline solution followed by ice-cold 4% PFA. The brains were removed and post-fixed for 4 hours in 4% PFA, followed by 20%, 30% sucrose until they sank. Coronal sections (30 μm) were collected using a cryostat (Leica) and stored in cryo-preservative solution at -20°C.

For immunofluorescence labeling, brain sections were subjected to free-floating immunohistochemistry, whereas neurosphere sections were subjected to slide-mounted immunocytochemistry. Sections were blocked with 10% donkey serum, 0.3% Triton X-100 for 1 hour at 37°C， and incubated with primary antibodies diluted in 5% donkey serum, 0.2% Triton X-100 for 2 hours at RT, and then transferred to 4°C overnight. Sections were incubated with appropriate secondary antibodies conjugated to Alexa fluor dyes, and mounted with Fluoromount-G™

(Southern Biotech). For DAB staining, sections were pre-treated with 1% H_2O_2 to eliminate endogenous peroxidase, incubated 48 hours with primary antibody, and then incubated with a biotinylated secondary antibody for 1 hour, followed by avidin-biotin-based peroxidase for 1 hour at RT to amplify the signal. DAB Substrate Kit (Vector Labs) was used to visualize the signal. RNA FISH experiments were performed using RNA-Scope reagents (Mm-Clstn2, Cat No. 542621; Hs-SLC17A6-O1, Cat No. 558401) and protocols (ACD Bioscience, CA) appropriate for fresh-frozen tissues. For Figure 6C and Figure 12E immunohistochemistry was performed following RNA FISH.

All antibodies used in this study are commercially available. The following antibodies were used in immunostaining: Mouse monoclonal anti-Human nuclei (MAB1281, Millipore, RRID: AB_94090), Mouse monoclonal anti-STEM121 (Y40410, Takara Bio Inc, RRID:AB_28013145), Mouse monoclonal anti-EN1 (4G11, DSHB, RRID: AB_528219), Goat polyclonal anti-FOXA2 (sc-6554, Santa Cruz Biotechnology, RRID: AB_2262810), Mouse monoclonal anti-synaptophysin (NBP1-19222, Novus, RRID: AB_1643135), Rabbit polyclonal anti-Calbindin D-28k (CB38, Swant, RRID: AB_2721225), Rabbit polyclonal anti-LMX-1 (AB10533, Millipore, RRID: AB_10805970), Sheep polyclonal anti-Tyrosine Hydroxylase (NB300-110, Novus Biologicals, RRID:AB_10002491), Rabbit polyclonal anti-Tyrosine Hydroxylase (P40101, Pel-Freez Biologicals, RRID: AB_2313713), Mouse monoclonal anti-Tyrosine Hydroxylase (T1299, Sigma-

Aldrich, RRID: AB_477560), Mouse monoclonal anti-Human NCAM (sc-106, Santa Cruz Biotechnology, RRID: AB_627128), Rabbit polyclonal anti-Trytophan hysroxylase 2 (NB100-74555, Novus, RRID:AB_2202792), Mouse monoclonal anti-Islet1 (40.2D6, DSHB, RRID:AB_528315), Rat monoclonal anti-DAT (MAB369, Millipore, RRID:AB_2190413), Rabbit polyclonal anti-PITX3 (ab30734, Abcam, RRID:AB_21653004), Rabbit polyclonal anti-GFAP (Z0334, Agilent, RRID:AB_10013382), Rabbit polyclonal anti-GABA (A2052, Sigma-Aldrich, RRID: AB_477652), Goat polyclonal anti-CHAT (AB144P, Millipore, RRID:AB_2079751), Rabbit polyclonal anti-Olig2 (Millipore, AB9610, RRID:AB_570666), Sheep polyclonal anti-human Collagen I alpha1 (AF6220, R&D, RRID:AB_10891543), Rabbit polyclonal anti-PTPRO (PA5-56964, Thermo Fisher Scientific, RRID:AB_2646135), Rabbit polyclonal anti-Serotonin (5-HT) (S5545, Sigma-Aldrich, RRID: AB_477522), Goat polyclonal anti-GIRK2 (ab65096, Abcam, RRID: AB_1139732), Rabbit polyclonal anti-HA (H6908, Sigma-Aldrich, RRID: AB_260070), Rat monoclonal anti-mCherry (M11217, Thermo Fisher Scientific, RRID: AB_2536611), Goat polyclonal anti-OTX2 (AF1979, R&D, RRID:AB_2157172), Rat monoclonal anti-GFP (04404-84, Nacalai Tesque, RRID: AB_10013361), Goat polyclonal anti-tdTomato (AB8181-200, SICGEN, RRID: AB_2722750), Donkey Anti-Mouse IgG (Alexa Fluor® 405) secondary (ab175658, Abcam, RRID:AB_2687445), Donkey Anti-Goat IgG (Alexa Fluor® 405) secondary (ab175664, Abcam, RRID:AB_2313502), Donkey anti-Mouse IgG (Alexa Flour 488) secondary (A-21202, Thermo Fisher Scientific,

RRID AB_141607), Donkey anti-Rabbit IgG (Alexa Flour 488) secondary (A-21206, Thermo Fisher Scientific, RRID:AB_2535792), Donkey anti-Rat IgG (Alexa Flour 488) secondary (A-21208, Thermo Fisher Scientific, RRID:AB_141709), Donkey anti-Sheep IgG (Alexa Flour 488) secondary (A-11015, Thermo Fisher Scientific, RRID:AB_141362), Donkey anti-Goat IgG (Alexa Flour 568) secondary (A-11057, Thermo Fisher Scientific, RRID:AB_142581), Donkey anti-Rabbit IgG (Alexa Flour 568) secondary (A-10042, Thermo Fisher Scientific, RRID:AB_2534017), Donkey anti-Mouse IgG (Alexa Flour 568) secondary (A-10037, Thermo Fisher Scientific, RRID:AB_2534013), Donkey anti-Rabbit IgG (Alexa Flour 647) secondary (A-31573, Thermo Fisher Scientific, RRID AB_2536183), Donkey anti-Mouse IgG (Alexa Flour 647) secondary (A-31571, Thermo Fisher Scientific, RRID:AB_162542), Donkey anti-Sheep IgG (Alexa Flour 647) secondary (ab150179, Abcam, RRID:AB_2884038), Donkey anti-Goat IgG (Alexa Flour 647) secondary (A-31573, Thermo Fisher Scientific, RRID:AB_2536183).

Imaging and quantification. All bright-field images (20x magnification) were captured using an Olympus VS120 microscope. Fluorescence images were taken on a Nikon TIE inverted microscope (60x magnification) and Olympus FV3000 microscope (20x magnification). For quantification of cultured neurospheres, the total number of Ho^+, TH^+, $LMX1A^+$, and $OTX2^+$ cells were counted from images obtained at 20x magnification to quantify the ratio of TH^+, $LMX1A^+$, and $OTX2^+$ cells. For graft

quantification, the total number of human nuclei (hN^+), TH^+, and $FOXA2^+$ cells within the grafts were counted from images obtained at 20x magnification to quantify the ratio of TH^+, or $FOXA2^+$ cells. For the percentage of $GIRK2^+$ or CB^+ DA neuron within grafts, TH^+ cells were first identified. To quantify other cell types in grafts, such as 5-HT^+, $GABA^+$, $OLIG2^+$, or $GFAP^+$, cells were co-labeled with TH and hN (except the GFAP group). Other-cell-types$^+$hN$^+$TH$^-$ were counted manually with ImageJ. $hCOL1A1^+$ fiber area within the graft was analyzed using ImageJ software. All data are presented as the mean ± SEM. To estimate graft volume, the section with immunostaining of hN was acquired at 20x magnification and analyzed with ImageJ. The graft area was extrapolated in every section of a 1:6 series, and the volume was calculated using Cavalieri's principle. To quantify mean gray value of tdT^+ fibers, all conditions consisting of staining and capturing sections were consistent. Four areas on the left and right side of the striatum were chosen, a threshold was set based on Li algorithm, and pixels within the threshold were quantified. Relative mean gray value was defined as mean gray value of grafted site minus the un-grafted site.

Generation of iPSCs from blood mononuclear cells. Twenty ml of venous blood was obtained from the donor after obtaining informed consent. Peripheral blood mononuclear cells (PBMCs) were isolated by standard density gradient centrifugation with Lymphoprep (Stemcell) at 400 g for 30 minutes. Cells were cultured in mononuclear cells (MNCs) medium containing 49% Ham's F-12 (Coring), 49% Iscove's modified Dulbecco's medium (IMDM, Gibco), 1% Insulin-Transferrin-

Selenium-Ethanolamine (ITSX, Gibco), 1% GlutaMAX (Gibco), 1% chemically defined lipid concentrate (Gibco), 200 μM 1-thioglycerol (Sigma), 50 μg/ml ascorbic acid, 5 mg/ml Bovine Serum Albumin (BSA, Sigma), and supplemented with 10 ng/ml recombinant human interleukin-3 (IL-3, PeproTech), 40 ng/ml human IGF-1 (PeproTech), 100 ng/ml recombinant human stem cell factor (PeproTech), 2 U/ml recombinant human erythropoietin (R&D), 1 μM dexamethasone (Sigma) and 100 μg/ml human holo-transferring (R&D). MNCs medium was used within 3 weeks. An approximately equal or greater number of cells over the starting cell population should be observed. Then, 2 million PBMCs were collected and electroporated with non-integrated vectors, including 3.3 μg pCXLE-hOCT34-shp53-F (Addgene), 3.3 μg pCXLE-hSK (Addgene), or 3.3 μg pCXLE-hUL (Addgene), using an Amaxa P3 Primary Cell Nucleofector Kit according to the standard protocol on 4D Nucleofector System (Lonza), program EO-100. After transfection, cells were resuspended in MNCs medium and plated onto a 12-well plate. Cells were incubated for 2 days at 37°C, 5% CO2. After 2 days of transduction and culture, transduced cells resuspended in MEF medium and plated at a density of 400,000 cells per well in a 6-well plate and then cultured on pre-seeded irradiated MEF feeder cells for reprogramming. On day 4 after transduction, hPSC medium containing 0.25 mM sodium butyrate (NaB, Sigma) was used throughout until clones were visible.

scRNA-seq of graft samples using the 10x genomics chromium platform. 3-month-old PD mice received striatal injections of mDA progenitors derived from surface marker-

tdT/AAVS1-CAG-EGFPnls-WPRE-polyA or AAVS1-CAG-EGFPnls-WPRE-polyA cell lines. Four months after transplantation, mice were deeply anesthetized, followed by transcardial perfusion with ice-cold oxygenated artificial cerebrospinal fluid (aCSF, in mM: 124 NaCl, 2.5 KCl, 1 NaH_2PO_4, 25 $NaHCO_3$, 37 glucose, 2 $CaCl_2$, 2 $MgSO_4$). The brain was removed, 200 μm vibratome sections were collected, and the graft (most in the striatum) was microdissected under a stereo fluorescence microscope with a cooled platform. The graft pieces were dissociated using a self-made ice-cold oxygenated dissection medium (DM, in mM: 81.76 Na_2SO_4, 120 K_2SO_4, 5.8 $MgCl_2$, 25.2 $CaCl_2$, 1 HEPES, 20 glucose, 20 NaOH) that contained 20 units/ml papain (Worthington), 1.1 mM EDTA, 0.067 mM 2-mercaptoethanol, 5.5 mM cysteine-HCl (Sigma), and 100 units/ml DNase I (Thermo Scientific), with 30-40 minutes enzymatic digestion, followed by manual trituration using fire-polished Pasteur pipettes and filtering through a 35 μm DM-equilibrated cell strainer. Cells were then pelleted at 400 g for 5 minutes. The supernatant was carefully removed. Cells were resuspended in 1-2 ml DM containing 2.5% Certified Fetal Bovine Serum (FBS, Biological Industries). Then, the debris was removed from the cell suspension using Debris Removal Solution (MiltenyiBiotec). Cell pellets were then resuspended in 200-400 μl DM containing 2.5% FBS for cell sorting, with the goal of enriching for EGFPnls-positive human cells for grafting. Chromium Single Cell 3' Reagent Kits (v3) were used for library preparation (10X Genomics). Libraries were sequenced on an Illumina Novaseq 6000.

Whole-cell patch-clamp recording of brain slice. Five months after cell transplantation,

coronal brain slices (300 mm thick) at the level of the forebrain were prepared from PD mice using a vibratome (Leica VT1200S) in an ice-cold cutting solution (in mM: 100 glucose, 75 NaCl, 26 NaHCO3, 2.5 KCl, 2 MgCl2-6H20, 1.25 NaH2PO4-6H20, and 0.7 CaCl2). Slices were transferred to recording artificial cerebrospinal fluid (aCSF, in mM: 124 NaCl, 4.4 KCl, 2 CaCl2, 1 MgSO4, 25 NaHCO3, 1 NaH2PO4, and 10 glucose) saturated with 95% O2 and 5% CO2 at 32ºC for 12 minutes, and then transferred into aCSF at room temperature. After 60 minutes of recovery, slices were transferred to a recording chamber and perfused continuously at 2-4 ml/minutes with oxygenated aCSF at 28ºC. Transplanted mDA neurons were identified by tdT fluorescence in the graft.

The initial access resistance was monitored throughout the experiment, ranging from 15-30 MΩ. Cells for which the access resistance changed >15% were discarded. Data were filtered at 1 kHz and digitized at 10 kHz. Voltage and current signals were recorded using an Axon 700B amplifier (Axon). The recording electrodes (3–5 MΩ) were filled with internal solution (in mM: 120 K+-Glucose, 5 NaCl, 0.2 EGTA, 10 HEPES, 2 MgATP, 0.1 Na3GTP, and 10 Phosphocreatine, adjusted to pH 7.2 with HCl) for action potential recording. Action potentials (APs) in response to depolarizing currents (0-180 pA, step 20 pA, duration 600 ms) were recorded in the current-clamp mood. Ramp current injections (100–300 pA, duration 2000 ms) were used to record the maximal discharging frequencies of mDA neurons. Voltage sag measurements were conducted under current-clamp mode by injecting current (-120 pA, duration 2000 ms) into the grafted mDA neurons.

The recording electrodes (3–5 MΩ) were filled with internal solution (in mM: 112 Cs-Gluconate, 5 TEA-Cl, 3.7 NaCl, 0.2 EGTA, 10 HEPES, 2 MgATP, 0.3 Na3GTP and 5 QX-314, adjusted to pH 7.2 with CsOH) for spontaneous excitatory post-synaptic current (sEPSC) and spontaneous inhibitory post-synaptic current (sIPSC) recording. For sEPSC or sIPSC recording, cells were voltage clamped at -60 or 10 mV, respectively.

Pre-processing of scRNA-seq data. scRNA-seq data were aligned with the human reference genome, GRCh38-3.0.0, and demultiplexed using the default parameters of Cellranger software (10x Genomics, v3.0.2 or v4.0.0). Obtained filtered count matrix was used for downstream analysis.

Clustering and identification of cell populations. The filtered count matrix was analyzed and processed using Seurat (v3.1.4) and Scanpy (v3.0.46), including data filtering, normalization, highly variable genes selection, scaling, dimension reduction, and clustering. First, scRNA-seq data sampled from each time point was created as Seurat object separately; genes with less than 3 counts were removed and cells with less than 200 genes detected were removed. Second, each Seurat object was converted into a loom file and imported into Scanpy for clustering. Then, six Seurat objects were merged using the "merge" function in Seurat and converted into a loom file to proceed with cell type clustering in Scanpy. Details of the downstream analysis are described as

the following:

Data filtering: cells with a mitochondrial gene ratio 5% were excluded. Then, cells with more than 1000 genes detected, less than 6000 genes detected (cells with more than 6000 genes detected are potential doublets), more than 1000 counts detected, and less than 40000 counts detected (cells with more than 40000 counts detected are potential doublets) were kept.

Data normalization: for each cell, counts were log normalized with the "NormalizeData" function in Seurat, "scale.factor" was set for 40000.

Highly variable genes selection: 2000 highly variable genes were calculated using the "FindVariableFeatures" function in Seurat. Then, we identified genes correlated with cell-cycle marker TOP2A (Pearson correlation greater 0.15) and excluded them from 2000 highly variable genes.

Cell cycle scoring: a cell-cycle related gene set with 43 genes expressed during G1/S and 54 genes expressed during G2/M was used to calculate an S phase score and a G2M phase score using the 'CellCycleScoring" function in Seurat. Cell cycle difference score was calculated as the difference value of S phase score minus G2M phase score.

Data scaling: the Seurat object was performed "ScaleData" function with default

parameters. Number of counts, number of genes, mitochondrial gene ratio, and the cell cycle difference score were variables to regress out in “ScaleData” function in Seurat.

Principal component analysis: highly variable genes were used to calculate principal components in the “RunPCA” function in Seurat. 100 principal components (PCs) were obtained and stored in Seurat object for computing neighborhood graph and umap in the next part.

Leiden clustering: Seurat object was converted into loom file and imported by Scanpy. A neighborhood graph of observations was computed by the “scanpy.pp.neighbors” function in Scanpy. Then, the leiden algorithm was used to cluster cells by “scanpy.tl.leiden” function in Scanpy.

Cluster merging and trimming: the top 200 differentially expressed genes for each cluster were calculated by the “scanpy.tl.rank_genes_groups” function in Scanpy using the parameters method = 'wilcoxon' and n_genes = 200. Cluster annotation was done manually based on previous canonical markers of developing midbrain and developing mouse brain atlas. For cell clusters that expressed similar marker genes, we merged them into one cluster; for cell clusters with unknown marker gene expression, we used Metascape, a web-based gene annotation analysis tool (3), to define their cluster identity. Combining filtered count matrix and analysis results of Metascape, we defined some cell clusters as low-quality cells (low counts and low genes detected), stressed,

apoptotic, high ribosome protein genes detected ratio, and hypoxic (Supplemental Table 4). Then, we filtered them out to obtain the trimmed cells list. Finally, we redid analysis steps 1-8 to get consistent clustering annotation for the separate time points and merged dataset.

Regional gene module score analysis. Regional gene modules were curated based on detectable genes in our datasets, previous research, and developing mouse brain atlas. Briefly, the mesencephalic gene module includes *OTX1*, *OTX2*, *LMX1A*, *EN1*, *PITX2*, and *SIM2*; the metencephalic gene module includes *HOXB-AS1*, *HOTAIRM1*, *HOXA2*, *HOXB2*, *GATA3*, and *GBX2*; MHB gene module includes *FGF8*, *FGF17*, *NKX2-8*, and *PAX8*. Then, the gene module score was calculated using the "scanpy.tl.score_genes" function in Scanpy. The regional cluster was assigned based on expression of the three gene modules on umap embeddings.

Single-cell RNA velocity analysis. First, we applied a command-line interface of velocyto (v0.17.17) to generate spliced/unspliced expression matrices for each time point scRNA-seq data (4). Then we combined all matrices in loom format and proceeded with downstream RNA velocity analysis using scVelo (v0.2.2) (5). We filtered out 21531 genes for which <20 counts were detected, kept the top 2000 highly variable genes, and took the intersection of cells used in clustering of merged time-course dataset. We recovered the whole splicing kinetics of 2000 highly variable genes, estimated velocities in 'stochastic' mode, and computed the velocity graph based on

cosine similarities. Stream plot of velocities was plotted on the umap embedding. A universal gene-shared latent time, which represents the internal clock of cells and is based only on its transcriptional dynamics, was computed based on velocity estimates in 'stochastic' mode and plotted on the umap embedding.

Pseudotime and gene cascade analysis. Only mDA-related clusters (0-P_MesenFP_LMX1A_Early, 1-P_MesenFP_LMX1A_Late, 2-P_vMesen_Stage II, 11-N_DA, 14-N_DA_Neuroblast) were extracted for pseudotime analysis. 1978 genes that were detected in less than 3 cells were filtered out. Then, data was processed similar to steps 2-6 of *Clustering and identification of cell populations*, including data normalization, highly variable gene selection, cell cycle scoring, data scaling, and principal component analysis. Pseudotime values were calculated using the URD package (v1.1.0) (6). Briefly, a diffusion map was calculated with the 'calcDM' function in URD using parameters knn = 200. The P_vMesen_Stage II cluster was chosen as root cells, then pseudotime was calculated by performing a probabilistic breadth-first search of the k-nearest neighbor graph that was used to generate the diffusion map calculated on the data. To find temporally varying genes that vary in pseudotime, we only considered genes expressed in at least 1% of cells as 'expressed genes'. Then, we calculated a spline curve that fit the mean expression of each group of five cells. We chose genes as temporally varying genes that: 1) change in their actual mean expression value by at least 0.5 and change in their scaled log2 mean expression value by at least 20%, 2) are well fit by the spline curves – here we set a threshold 0.045

on the sum of squared residuals, 3) are fit by the spline curve significantly better than a flat line of slope 0 – here we set a threshold 0.25 as a better fit than flat. Next, we took the intersection of those genes and used them in the gene expression cascade analysis. We used Metascape (http://metascape.org) to perform gene ontology (GO) analysis of each gene cluster.

For ligand-receptor cascade, we filtered varying ligands and receptors from three crucial signaling pathways involved in mDA neuron differentiation based on public data resources (SHH, FGF, and WNT pathways) (7). In total, we obtained 3 varying ligands or receptors in the SHH pathway (ligand: GNAS and SHH; receptor: PTCH1), 4 in the FGF pathway (ligand: NCAM1; receptor: SDC2, FGFR1, and CDH2), and 4 in the WNT pathway (ligand: WNT5A, RSPO2, and SFRP1; receptor: FZD1). Then we used these genes to calculate cascade expression.

Cell type replicability analysis. To evaluate the similarity between cell types generated in vitro and cells that developed in vivo in the human midbrain, we applied the MetaNeighbor R package (v1.6.0) to calculate the AUROC score as a performance vector on neuron types and progenitor types. First, we chose 2000 genes as integration features of our Seurat object and public datasets prepared as Seurat object. Then, we integrated two datasets using anchors found by the 'FindIntegrationAnchors' function. Next, we prepared a normalized data matrix of 2000 anchor genes (variable genes) as SummarizedExperiment class using the SummarizedExperiment R package (v1.16.1).

Fast, low memory, and unsupervised versions of MetaNeighbor were used to calculate the AUROC score (MetaNeighborUS function, fast version was implemented). Cross-dataset mean AUROC scores were plotted in the heatmap.

Integration of graft scRNA-seq datasets. For integrating analysis of graft sample scRNA-seq datasets, we applied Harmony (v1.0) (8) integration to reduce technical batch effects between different marker-sorted groups (two batches for the unsorted group, one for the CLSTN2-derived group, and one for the PTPRO-derived group). The 'RunHarmony' function in the Harmony package was used to calculate corrected Harmony coordinates. Then, the Seurat object was converted into a loom file and imported by Scanpy. A neighborhood graph of observations was computed by the "scanpy.pp.neighbors" function in Scanpy. Then, the leiden algorithm was used to cluster cells by the "scanpy.tl.leiden" function in Scanpy, which used the corrected Harmony embeddings rather than PCs. Cluster annotation was done manually based on previous canonical markers of major cell types. For further clustering of neurons, we filtered neuron clusters examined by *STMN2* expression. A total of 2736 neurons were extracted, but 1277 stressed cells were filtered out, and we repeated data processing similar to steps 2-6 of *Clustering and identification of cell populations*.

Certain gene-positive cell ratio and averaged pseudo-bulk expression estimation across stages. To estimate the percentage of certain gene representative cell populations, we randomly selected 10% of cells from each timepoint scRNA-seq

dataset, and repeated 10 times as 10 trials. Then we set a threshold for UMI counts of certain genes (number of UMI > 0). Finally, we designated cells that had higher UMI counts than the threshold as gene-positive cells. For averaged pseudo-bulk expression, we averaged gene expression by stages of merged time-course scRNA-seq datasets using 'AverageExpression' in Seurat. Then chosen genes (*LMX1A*, *EN1*, *CLSTN2*, and *PTPRO*) averaged expression was extracted and plotted.

Estimation of cell type proportions in bulk RNA-seq data using scRNA-seq data. To perform deconvolution analysis, we used MuSiC (v0.1.1) (9) to estimate cell type proportions in bulk RNA-seq data using scRNA-seq data derived from the corresponding stage (stage III and stage IV). Both bulk and scRNA-seq datasets were prepared as 'ExpressionSet' objects using the Biobase package (v2.46.0). Input gene markers from each scRNA-seq cell type were extracted from the top 500 DEGs calculated by the 'FindAllMarkers' function in Seurat using default one-tailed Wilcoxon rank-sum test.

Bulk RNA-seq sequencing and data analysis. For transcriptional analysis of the LMX1A-tdT/EN1-mNeonGreen reporter line, cell lines were prepared to differentiate into mDA neurons as in *Generation of midbrain dopamine neurons*. Cells were digested as described above and sorted by BD LSRFortessa flow cytometer or MA900 Multi-Application Cell Sorter at stage III or IV. $tdT^{+}Neongreen^{+}$ cells were collected as one group, whereas $tdT^{-}Neongreen^{+}$, $tdT^{+}Neongreen^{-}$, and $tdT^{-}Neongreen^{-}$ cells were

collected together as another group. Cells were pelleted at 400 g for 5 min and lysed with TRIzol (Thermo Scientific). Sequencing libraries were generated using NEBNext UltraTM RNA Library Prep Kit for Illumina (NEB, USA) and index codes were added to attribute sequences to each sample. Libraries were sequenced on an Illumina Hiseq PE150. Raw sequencing reads were processed by quality control and adaptor trimming. Then, clean sequencing reads were mapped to the UCSC human GRCh38 genome with HISAT2 software (v2.1.0). Bam files were generated using Samtools (v1.7). Reads were counted by the 'summarizeOverlaps' function in the GenomicFeatures R package (v1.38.2). Differential expression analysis was performed using the 'glmTreat' function in edgeR R package (v3.28.1). DEGs were chosen with an absolute fold change of >1.5 (|Log2FC| > 1.5) and a cutoff Benjamini-Hochberg adjusted p-value of 0.05. For the DEGs volcano plot, the chosen cutoff for statistical significance was 0.001, and chosen cutoff for absolute log2 fold-change was 2. Volcano plots were implemented using the EnhancedVolcano R package (v1.4.0).

References

1. Xiong M et al. Human Stem Cell-Derived Neurons Repair Circuits and Restore Neural Function.. *Cell Stem Cell* 2021;28(1):112-126.e6.

2. Chen Y et al. Chemical Control of Grafted Human PSC-Derived Neurons in a Mouse Model of Parkinson's Disease. *Cell Stem Cell* 2016;18(6):817–826.

3. Zhou Y et al. Metascape provides a biologist-oriented resource for the analysis of systems-level datasets. *Nat. Commun.* 2019;10(1):1523.

4. La Manno G et al. RNA velocity of single cells. *Nature* 2018;560(7719):494–498.

5. Bergen V, Lange M, Peidli S, Wolf FA, Theis FJ. Generalizing RNA velocity to transient cell states through dynamical modeling. *Nat. Biotechnol.* 2020;38(12):1408–1414.

6. Farrell JA et al. Single-cell reconstruction of developmental trajectories during zebrafish embryogenesis. *Science* 2018;360(6392).

7. Ramilowski JA et al. A draft network of ligand-receptor-mediated multicellular signalling in human. *Nat. Commun.* 2015;6:7866.

8. Korsunsky I et al. Fast, sensitive and accurate integration of single-cell data with Harmony. *Nat. Methods* 2019;16(12):1289–1296.

9. Wang X, Park J, Susztak K, Zhang NR, Li M. Bulk tissue cell type deconvolution with multi-subject single-cell expression reference. *Nat. Commun.* 2019;10(1):380.
